# Application of Novel Polymer Materials Containing Deep Eutectic Solvents for the Separation of Metal Ions from Alkaline Battery Leachates

**DOI:** 10.3390/ma18122768

**Published:** 2025-06-12

**Authors:** Daria Bożejewicz, Małgorzata A. Kaczorowska

**Affiliations:** Faculty of Chemical Technology and Engineering, Bydgoszcz University of Science and Technology, Seminaryjna 3 Street, 85-326 Bydgoszcz, Poland; daria.bozejewicz@pbs.edu.pl

**Keywords:** polymer materials, adsorption, deep eutectic solvents, ionic liquids, metal ions, battery leachates

## Abstract

The widespread, worldwide utilisation of alkaline batteries requires development of proper recycling methods for used batteries, which are considered both as a secondary source of valuable metals and as a threat to the environment (may contain toxic substances). As many separation methods of metal ions from battery leachates are based on the use of substances that require complex synthesis or are not eco-safe, new materials suitable for this purpose are systematically sought. Therefore, in this study, the results of the separation of Ni(II), Zn(II) and Mn(II) ions from alkaline battery leachates using polymer materials (PMs) impregnated with easily synthesised, “green” deep eutectic solvents (DESs) or with ionic liquids (ILs) were presented. Additionally, PMs surface wettability were determined and their chemical compositions were analysed using the Fourier transform infrared spectroscopy–attenuated total reflectance (FTIR–ATR) method. Among all PMs synthesised, materials containing DESs (composed of Aliquat 336 or Cyphos IL 101 and diacetamide) performed best in the separation of Ni(II) ions (removal of 93.42% and 80.86%). The application of DES-based PMs for the separation of metal ions from battery leachates is in line with green chemistry principles, and such materials can potentially be used in the processing of e-waste.

## 1. Introduction

The widespread use of primary alkaline (AB) and zinc–carbon (ZCB) batteries in various types of portable household electronic devices (e.g., radios, toys, pocket lamps, cameras, etc.) and their relatively short service life leads to the generation of significant amounts of battery waste. Due to the composition of such batteries, AB and ZCB waste may contain both valuable elements (e.g., Zn, Mn), which should be recovered to sustain a circular economy, and substances that, if released into the environment, could pose a threat to living organisms (e.g., Pb(II)). The need to properly manage (e.g., collect and recycle) used alkaline and zinc–carbon batteries is therefore necessary for both economic and environmental reasons [1]. The development of efficient and environmentally safe methods of waste battery treatment, consistent with the principles of sustainable development and resource management, is also necessary due to the forecasted increase in the importance of batteries (e.g., as a result of technological progress, increased interest in mobile devices, etc.), their even wider use in the future and consequently the generation of more waste of this type [2]. The global battery market is expected to continue to grow steadily, e.g., in the case of rechargeable batteries, it is expected to reach approximately USD 195 billion by 2032, with an annual growth rate of over 5% (relative to the years 2023–2032) [3]. Issues related to recycling of used batteries are so crucial that they have been included in various legal acts, e.g., the European Union (EU) Batteries Regulation sets policies that enforce the use of recycled materials from spent batteries in new batteries (to facilitate circular production, to limit greenhouse gases emissions from the extraction and processing of the materials, etc.) [4,5]. The need to recycle used batteries is closely related to the growing interest in development of efficient and eco-safe methods intended for separation of various materials from this type of waste. The recovery of valuable components (e.g., metal ions) from used batteries can be carried out using various processes, but the most common are pyrometallurgical and hydrometallurgical methods. The application of pyrometallurgical methods based on high-temperature smelting is associated with several limitations such as, for example, the consumption of large amounts of energy and usually with the generation of a certain amount of toxic gases, and it leads to the formation of alloys. In hydrometallurgical processes, used battery electrode material (free of plastic and paper, obtained after shredding, attrition, magnetic separation of ferrous materials, etc.) is leached using acids, alkalis, or complex leaching liquids, often with the addition of oxidising agents, and then metal ions are recovered using one or more of available separation methods (e.g., chemical precipitation, filtration, extraction, membrane processes, etc.) to obtain a high-purity recycled product [6,7]. Limitations of hydrometallurgical methods may include the complexity of procedures to which used batteries must be subjected prior to separation (involving labour and energy inputs) and the need to use various, often toxic substances that may have a negative impact on the environment. However, the efficiency of recovering of specific metal ions from battery leachates depends on many factors, including the leaching method and chemical reagents used, the properties of the separated metal ions, the composition and properties of the battery waste, and experimental conditions [7]. In general, batteries can be divided into several groups (e.g., primary batteries, secondary batteries, battery systems for grid-scale energy provision, fuel cells, and electrochemical capacitors (supercapacitors)) [8]. Primary batteries include alkaline batteries consisting of a negative zinc electrode, a positive manganese dioxide (MnO_2_) electrode, and potassium hydroxide (alkaline electrolyte), and zinc–carbon batteries contain a zinc anode, a carbon cathode, and ammonium chloride (acidic electrolyte) [7,8]. Typically, hydrometallurgical methods enable the recovery of significant amounts of zinc and manganese from used alkaline batteries [9,10], or zinc in the case of zinc–carbon batteries [11]. Additionally, it has been shown that under optimal leaching and separation conditions, the recovery of other metal ions (e.g., iron, cadmium, chromium, cobalt, copper, nickel, or potassium) can also be successfully performed [12,13]. However, due to a number of factors that affect the efficiency of the hydrometallurgical recovery of various metal ions from battery waste, determining the optimal conditions is not easy, and in addition, attention must be paid to ensuring that the process is not only efficient, but also cost-effective and as environmentally friendly as possible [9,14]. Conventional methods often do not meet all the requirements of economic and environmental sustainability. In order to achieve these goals, research is currently being conducted to modify both the methods of leaching battery waste (e.g., by the application of multi-stage leaching using different chemicals or microwave-assisted reducing acid leaching) and the utilisation of new solutions (which are more economical and eco-friendly) enabling the efficient separation of metal ions from battery leachates [13,14,15]. In the field of separation methods, studies are being undertaken, among others, on the possibility of using more “green” (comparing to traditionally utilised) substances that have the ability to bind metal ions (e.g., ILs, DESs) and on the possibility of application of various polymer membranes for the recovery of metal ions from leachates (mainly lithium ions from lithium ion battery leachates), because membranes can be relatively easily modified by changing their components (quantitatively and qualitatively), and membrane-based processes are considered environmentally friendly [16,17]. In this context the possibility of using non-toxic DESs and ILs in membrane processes intended for recovery of metal ions from battery leachates seems to be particularly promising in relation to environmental protection.

In general, membrane technology shows significant advantages over traditional separation methods, such as solvent extraction, because the formation of membranes does not generate significant production costs and does not require the use of toxic chemical compounds. The application of membranes allows for easy scaling and reduces negative environmental impacts. However, the membrane properties depend on the casting conditions, the type and weight percentage of polymer matrix used, as well as the additives, e.g., plasticiser and carrier [18]. Deep eutectic solvents as binding compounds for various contaminants have found specific applications in membrane technology used to recover metal ions, e.g., from batteries. Deep eutectic solvents, which are considered a “greener” alternative to ionic liquids, have properties similar to ILs, such as low volatility and high thermal stability. However, DESs are also biodegradable, non-toxic, and usually cheaper than ILs. Additionally, they can be obtained by a simple synthesis and show a lower melting point than their constituents [19,20,21]. Ionic liquids are pure compounds, unlike DESs, which are mixtures of compounds that serve as hydrogen bond donors (HBDs) or hydrogen bond acceptors (HBAs). In general, depending on HBAs and HBDs, DESs are classified into five types: (type-I) DESs that consist only of ionic compounds; (type-II) DESs that usually constitute a mixture of salts and metal chloride hydrates; (type-III) DESs that consist of salts and molecular compounds; (type-IV) DESs that are mixtures of metal chlorides with neutral organic molecules; (type-V) DESs that consist of two molecular compounds [22,23]. Deep eutectic solvents have been successfully used in polymer membranes to remove metal ions from aqueous solutions. Depending on the composition of polymer membranes containing DESs, the PMs allow the removal of gold(III), nickel(II), and cobalt(II) ions [24,25]. In recent years, deep eutectic solvents have also been used in the separation of metal ions from battery leachates. For example, DES composed of TOPO and decanoic acid was used to selectively precipitate nickel(II) ions (90%) from a leaching solution consisting of Co(II), Mn(II) and Cu(II) ions [26]. Another DES, a mixture of H_3_PO_4_ and choline chloride, enabled efficient extraction of Li(I) and Co(II) ions from battery leachate, with extraction efficiency of 94.3% and 80.8%, respectively, after five consecutive cycles [27]. As can be seen from the examples given, various chemical compounds have been used for preparation of deep eutectic solvents intended for metal ion separation processes. We recently showed that diacetamide, a chemical compound with well-known metal ion-binding properties, can also be used for preparation of a DES (containing diacetamide and Aliguat 336)-based polymer adsorptive membrane, efficient in Cu(II) and Zn(II) ion removal from computer scrap leachates [28]. However, the potential use of this compound for the preparation and application of DES-based PMs has not yet been well explored.

**Figure 1:**
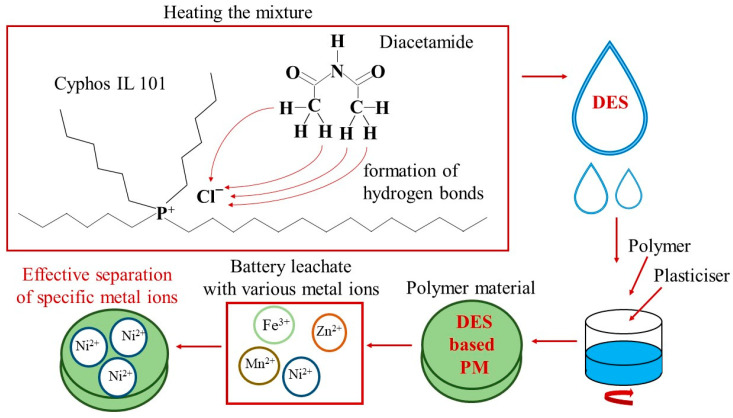
A simplified scheme of the preparation and operation of polymer material containing DES made from Cyphos IL 101 and diacetamide.

Therefore, in this article, we present the results of the synthesis and characterisation of novel polymer materials containing deep eutectic solvents composed of diacetamide as HBA and Cyphos IL 101 or Cyphos IL 104 (or Aliquat 336, for comparison) as HBD and their first application for the removal of Zn(II), Ni(II), and Mn(II) ions from real solutions obtained by leaching black mass (anode and cathode material) of primary alkaline batteries. Figure 1 shows a simplified scheme of the preparation and operation of polymer material containing DES made from Cyphos IL 101 and diacetamide. Additionally, the results of metal ion separation using polymer materials containing DESs or ILs (Cyphos IL 101, Cyphos IL 104, or Aliquat 336) are compared. They indicate that PMs with DESs composed of Aliquat 336 or Cyphos IL 101 and diacetamide enabled efficient separation of nickel(II) ions from battery leachate. Moreover, the possibility of application of DES-based PMs considered safe for the environment for the effective separation of metal ions from solutions obtained by leaching battery waste is in line with the sustainable development trend.

## 2. Materials and Methods

### 2.1. Chemical Reagents

Concentrated chloric and nitric acids used for the preparation of aqua regia (by mixing of acids in a volume ratio of 3:1, respectively) were purchased from Avantor (Gliwice, Poland). The ionic liquids Cyphos IL 101 and Cyphos IL 104, as well as diacetamide, were purchased from Sigma-Aldrich (Poznan, Poland), whereas Aliquat 336 was obtained from Thermo Scientific (Waltham, MA, USA). Poly(vinyl chloride) (PVC) of an average molecular weight of 72,000, was purchased from Anwil (Wloclawek, Poland); bis(2-ethylhexyl) adipate (ADO) and tetrahydrofuran were acquired from Avantor (Gliwice, Poland). All reagents were of analytical grade and were used without further purification.

### 2.2. Primary Alkaline Battery Preparation

**Figure 2:**
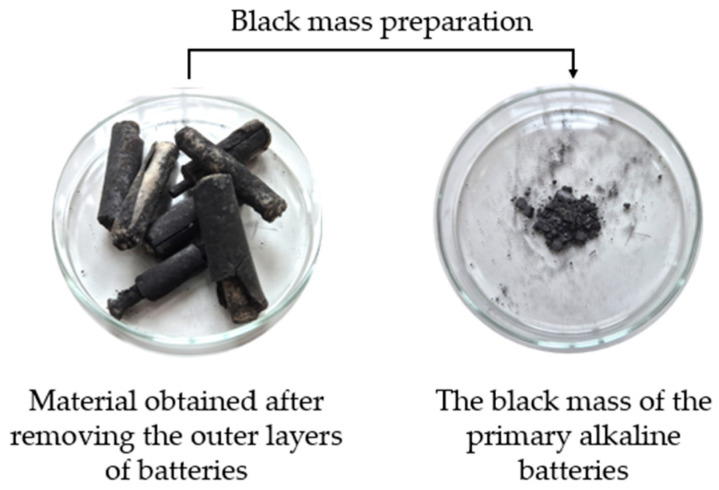
Material obtained after removing the outer layers of batteries (**left**), the black mass of used primary alkaline batteries (**right**).

The outer layer of the used primary alkaline batteries of AA type (presented in Figure 2), purchased from the local market, was removed and the black mass containing the anode and cathode material was separated and thoroughly mechanically crushed. The obtained powder (1.5 g) was leached with an aqua regia solution (150 mL) to enable fast and efficient transfer of metal ions to the aqueous phase, in a static process at a temperature of 20 ± 2 °C. The resulting solution was filtered and analysed using atomic absorption spectrometry (AAS 240FS Spectrometer, Agilent, Santa Clara, CA, USA) to determine metal ion content. The initial concentrations of nickel(II), manganese(II), and zinc(II) ions in solution obtained after leaching the battery black mass were 0.035 mg/L, 0.49 mg/L, and 53.59 mg/L, respectively. Subsequently, separation processes were carried out using the synthesised polymer materials.

### 2.3. Preparation of Deep Eutectic Solvents

Deep eutectic solvents containing diacetamide as a hydrogen bond donor and one of the ionic liquids (i.e., Cyphos IL 101, Cyphos IL 104, or Aliquat 336) as a hydrogen bond acceptor were prepared by mixing the components in a 2:1 molar ratio, according to the procedure described earlier [28]. Table 1 provides detailed information on the composition of the DESs produced. All obtained DESs were liquid at room temperature. Next, ^1^H NMR and ^13^C NMR spectra of all produced DESs were obtained using a Bruker Avance III 400 MHz spectrometer (Bruker, Billerica, MA, USA) (with DMSO-d6 solution).

### 2.4. Preparation of Polymer Materials

For the preparation of all polymer materials (PM-1 to PM-6), solutions containing about 60 wt.% of poly(vinyl chloride) as the polymer base, about 20 wt.% of bis(2-ethylhexyl) adipate as the plasticiser, and about 20 wt.% of specific chelating agent (DES-1, DES-2, DES-3, Cyphos IL 101, Cyphos IL 104, or Aliquat 336) were prepared in 10 mL of tetrahydrofuran. A solution containing only PVC and ADO was also prepared (PM-0). Next, each solution was poured onto a glass ring (having a diameter of 4.5 cm) and the solvent was allowed to evaporate (ambient air, temperature of 20 ± 2 °C). After 24 h, when THF completely evaporated, the obtained PMs were separated and conditioned with distilled water for 24 h. Thus, homogeneous and flexible polymer materials were obtained. Table 2 shows the composition of the produced polymer materials.

### 2.5. Characterisation of the Surface of the Prepared Polymer Materials Before Sorption Processes

FTIR-ATR analysis: The structures of all obtained PMs (PM-0 to PM-6) were analysed using Fourier transform infrared spectroscopy-attenuated total reflectance (FTIR-ATR) method, with a Bruker Alpha-PFT-IR device (Bruker, Billerica, MA, USA). For each membrane, FTIR-ATR spectra were recorded in the wavenumber range of 400–4500 cm^−1^.

Wettability analysis: The static water contact angles on the surfaces of the obtained PMs were measured by the sessile drop method using a contact angle goniometer (T-1, DSA100E—Drop Shape Analyser, KRÜSS Scientific, Hamburg, Germany). All experiments were performed at a temperature of 23 °C. After thoroughly washing with distilled water and drying with filtration paper to remove moisture, the synthesised PMs were examined by placing 5 μL droplets of deionised water on the surfaces of the polymer materials and measuring CAs within 3 s of adding the drops. The average values of CAs (based on measurements at three random locations) were reported.

### 2.6. Separation Processes

Seven separation systems were prepared by dilution of 15 mL of the previously prepared battery leachate solution with distilled water to obtain 30 mL solutions. Next, the synthesised polymer materials (PM-0 to PM-6) were immersed in the obtained solutions for 1 hour (static process at a temperature of 20 ± 2 °C). The metal ion concentrations in the aqueous phases after separation processes were determined by atomic absorption spectrometry (AAS 240FS Spectrometer, Agilent, Santa Clara, CA, USA).

### 2.7. Characterisation of Polymer Materials After Sorption Processes by Scanning Electron Microscopy—Energy-Dispersive Spectroscopy (SEM-EDS)

The synthesised polymer materials (PM-0 to PM-6) after sorption processes were analysed by scanning electron microscopy—energy-dispersive spectroscopy (SEM-EDS) using scanning electron microscope with focused ion beam (ZEISS EVO 10 Scanning Electron Microscope, ZEISS, Oberkochen, Germany).

## 3. Results and Discussion

### 3.1. Composition of Synthesised DESs

The composition of the synthesised deep eutectic solvents was confirmed by nuclear magnetic resonance spectroscopy (^1^H NMR and ^13^C NMR). The ^1^H NMR and ^13^C NMR spectra of DES-1 and DES-2, obtained using a Bruker Avance III 400 MHz spectrometer (with DMSO-d6 solution), are presented in Supplementary Materials in Figures S1A,B and S2A,B. Additionally, for comparison, ^1^H NMR spectra of the individual components of DESs (i.e., diacetamide, Cyphos IL 101, Cyphos IL 104) are provided in Supplementary Materials in Figure S3a–c. The results of the analysis of ^1^H NMR and ^13^C NMR spectra obtained for deep eutectic solvent DES-3 were described earlier [28]. Based on the results obtained from NMR experiments, it is not possible to clearly determine the nature of the connections between diacetamide (HBD) molecules and the ions of ionic liquids (acting as HBAs). However, taking into account previously published results, it can be assumed that hydrogen interactions play a key role in formation of DESs [28,30,31].

### 3.2. Characterisation of Polymer Materials Before Sorption Processes

#### 3.2.1. Contact Angle Measurement of PMs

The results of the contact angle (CA) measurements of the synthesised polymer materials PM-0 to PM-4 are presented in Table 3. The data related to the measurement of CAs of PM-5 (containing PVC, ADO, and DES-3) and PM-6 (containing PVC, ADO, and Aliquat 336) have been published earlier (PM-5 and PM-6 had CAs of 48.8° and 7.3°, respectively) [28]. All experiments were performed under the same conditions. Based on the determined contact angles, it has been concluded that the surfaces of all analysed polymer materials are hydrophilic—the CAs were less than 90° in all cases. It is evident that contact angles strongly depend on the composition of polymer materials. The CA value of PM-0, which contains only PVC and ADO, was 74.42°. PM-1, which additionally contains DES-1, had a slightly higher CA of 78.9°. For all other polymer materials, the CA values were lower than that of PM-0. The lowest CA values were measured for PM-4 and PM-6, which contain ILs (Cyphos IL 101 or Aliquat 336, respectively). The obtained results of CAs indicate that by changing only one component of a polymer material, its physical properties can be relatively easily modified, which may influence its separation performance. Based on literature data, it can be concluded that PVC is a slightly hydrophobic polymer (CA of 76°), and the addition of a plasticiser reduces the contact angle [32]. Adding a plasticiser to PVC facilitates increased transport of metal ions and makes the polymer material more flexible and soft (although an excess of plasticiser may reduce membrane performance) [33]. In general, according to the absorption–diffusion mechanism, good hydrophilicity of the absorbent enhances water wetting during the adsorption process. Hydrophilic adsorption materials have surfaces that are easily wetted by water, allowing the pollutants (e.g., metal ions, synthetic dyes) contained in aqueous solutions to be contacted and adsorbed efficiently [34]. However, it should be emphasised that hydrophilic/hydrophobic properties of polymer materials are only one of several factors influencing the efficiency of the separation process.

#### 3.2.2. FTIR-ATR Analysis of Synthesised PMs

FTIR-ATR spectroscopy was used to detect the frequencies of the functional groups present in the synthesised polymer materials and consequently to gain more information regarding their composition (about the presence of individual components and the manner in which they bond). All obtained FTIR-ATR spectra of PMs, from PM-0 to PM-2, PM-5, and PM-6, are presented in Supplementary Materials in Figures S4–S8. FTIR-ATR spectra of polymer materials PM-3 and PM-4, which contain Cyphos IL 101 and Cyphos IL 104, respectively, were characterised in a previously published work [35]. FTIR-ATR spectrum of PM-0 (Figure S3) shows characteristic bands related to functional groups originating from its two components, PVC and ADO. For example, the bands from 2862.51 to 2928.80 cm^−1^ can be attributed to the bending modes of –CH_2_ and –CH_3_ bonds present in both components and to the stretching vibration of the alkane group (–CH_2_–CH_3_) present in ADO structure. The bands at 1250.82 and 1332.12 cm^−1^ and in the range from 609.09 to 691.27 cm^−1^ may correspond to the stretching modes of C–Cl bonds (present in PVC) and to the bending modes of C–H bonds (present in both components of PM-0), respectively. Characteristic bands associated with the use of ADO in PM-0 synthesis also include a band at 1725.66 cm^−1^ and bands in the range of 1004.22–1250.82 cm^−1^, which may correspond to the stretching mode of the C=O group and to the ester group, respectively. In FTIR-ATR spectra of polymer materials containing, apart from PVC and ADO, the appropriate DESs or ILs, in addition to the signals corresponding to the functional groups originating from the polymer and the plasticiser, additional signals related to the presence of deep eutectic solvents or ionic liquids are visible. For example, characteristic bands at about 1629 cm^−1^, 1644 cm^−1^ and 1639 cm^−1^ in the spectra obtained for PM-1, PM-2, and PM-5, respectively, may be related to the amide group originating from diacetamide. The bands at about 1250 cm^−1^ visible in FTIR-ATR spectra of PM-1, PM-2, PM-3, and PM-4 may be related to the P=O groups from Cyphos IL 101 or Cyphos IL 104 (which were used to obtain DESs for PM-1 and PM-2, respectively, and are directly included in PM-3 and PM-4) [36]. In FTIR-ATR spectra of PM-5 and PM-6 materials, in addition to the frequencies corresponding to characteristic functional groups originating from PVC and ADO components, bands at about 1354–1251 cm^−1^ are observed, which can be assigned to the amino group of Aliquat 336 (which serves as a substrate for DES-3 in PM-5 and is a direct component of PM-6). All these characteristic bands visible at PMs FTIR-ATR spectra allow to confirm the presence of individual components of the synthesised polymeric materials (PVC, ADO, specific ILs or DESs) and assume that these components are connected mainly via hydrogen bonds and/or van der Waals interactions. Based on the analysis of the spectra, the formation of covalent bonds cannot be confirmed, which is consistent with previous results [35].

### 3.3. Sorption Processes

**Figure 3:**
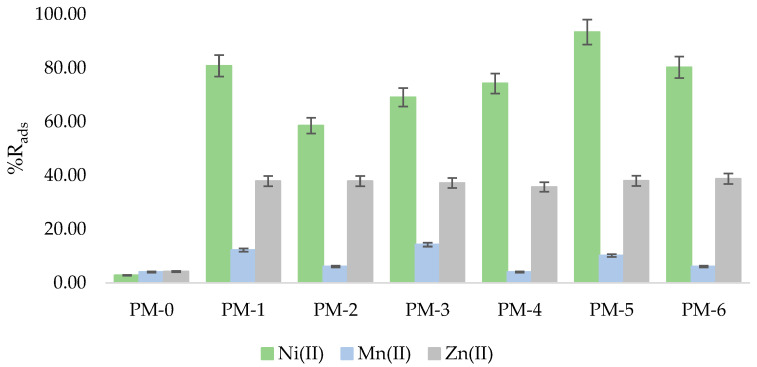
Metal ion sorption efficiencies of synthesised polymer materials, PM-0 to PM-6, after a 1 h sorption process conducted in an aqua regia leachate of alkaline battery black mass. The average %R_ads_ in all experiments was ±0.01.

To assess whether the synthesised polymer materials can be used for the separation of metal ions from solutions obtained by leaching battery black mass, basic parameters of removal efficiency were determined, such as efficiency of sorption (%R_ads_) and sorption capacity (q_t_). Metal ion sorption efficiency is the percent of metal ion-binding by the adsorbent, and sorption capacity is the mass of metal ions, in grams, adsorbed per gram of the adsorbent. %R_ads_ and q_t_ were calculated based on the literature [37]. Figure 3 shows metal ion sorption efficiencies of synthesised polymer materials (PM-0 to PM-6) for nickel(II), manganese(II), and zinc(II) ions after a 1 h sorption process conducted in acidic leachate from black mass. Table 4 presents sorption capacities of the investigated PMs.

Based on the obtained results of sorption of Ni(II), Mn(II), and Zn(II) ions from alkaline battery leachates, it can be concluded that all the synthesised polymer materials containing ILs or DESs were the most effective in the separation of nickel ions. The highest R_ads_ values for Ni(II) ions (after 1 h of the sorption process) were obtained using PM-5 and PM-1 containing DES-3 (composed of Aliquat 336 and diacetamide) and DES-1 (composed of Cyphos IL 101 and diacetamide), respectively (93.42% and 80.86%), and applying PM-6 containing Aliquat 336 ionic liquid (80.29%). The PM-2 containing DES-2 (composed of Cyphos IL 104 and diacetamide) was less efficient in nickel ion separation (%R_ads_ = 58.57%) than the PMs containing ionic liquids Cyphos IL 101 or Cyphos IL104 (%R_ads_ were 69.14% and 74.29%, respectively). The efficiency of the synthesised PMs (from PM-1 to PM-6) in relation to zinc(II) ions was similar, regardless of the type of polymer material used, and ranged from 35.73% (PM-4) to 38.77% (PM-6). In the case of Mn(II) ion separation, the %R_ads_ values obtained using different PMs were more diverse than in the case of zinc(II) ion removal, but significantly lower. The highest %R_ads_ value for Mn(II) ion separation was obtained using PM-3 containing Cyphos IL 101 (14.29%) and the lowest was recorded using PM-4 with Cyphos IL 104 (4.08%). The efficiency of PM-0 containing only polymer and plasticiser with respect to all separated metal ions was very low (2.86% for Ni(II), 4.08% for Mn(II) and 4.29% for Zn(II) ions, respectively). The obtained results clearly indicate that the appropriate selection of polymer material components, especially chemical compounds that are able to bind metal ions (e.g., ILs, DESs), has a significant impact on the properties of PMs and their efficiency in separation process. However, the properties of the separated metal ions and their affinity towards specific ILs and DESs are also important. Of all the metal ions analysed, nickel was removed most effectively by all the adsorption materials, followed by zinc, and the least effective were the manganese removal processes. This relationship is probably influenced by the stability of the complexes formed and the difference in the ionic radii of the metal ions (which is in the order Ni^2+^ (72 pm) > Zn^2+^ (74 pm) > Mn^2+^ (80 pm)). The stability of the metal ion complexes is closely related to the size of the ionic radius of the metal ions involved. Moreover, it was reported in the case of many adsorbents that the smaller the ionic radius of the bounded metal ion, the higher the adsorption affinity [38,39,40,41]. The sorption capacity of all synthesised polymer materials after a 1 h sorption process was the highest for zinc ions, which is related to the nature of the solution used (alkaline battery leachate containing significant amounts of zinc ions). To confirm the sorption performance of the polymer materials tested, the PMs yielded from the separation processes were subjected to SEM-EDS analysis.

### 3.4. SEM-EDS Characterisation of PMs After Sorption Experiments

**Figure 4:**
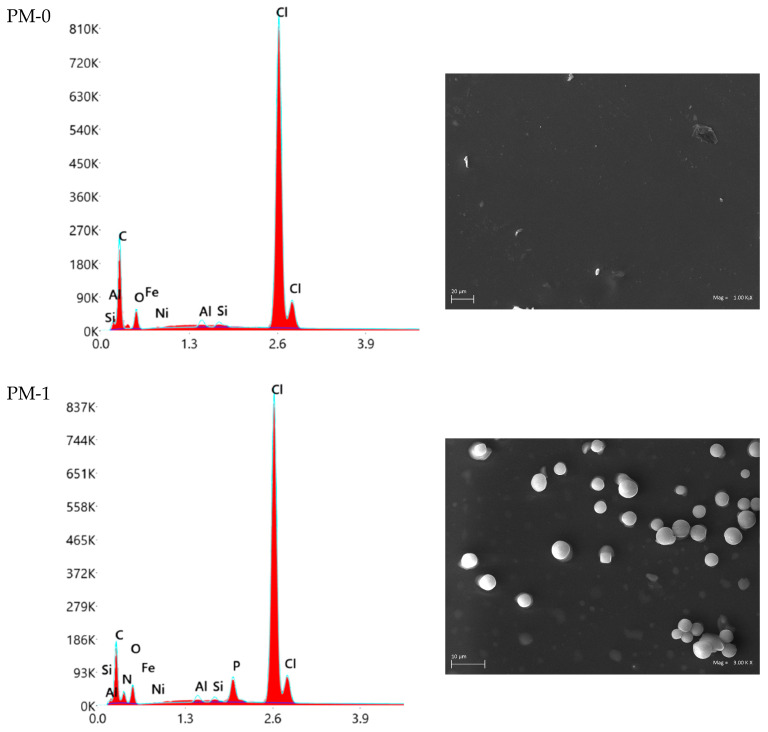

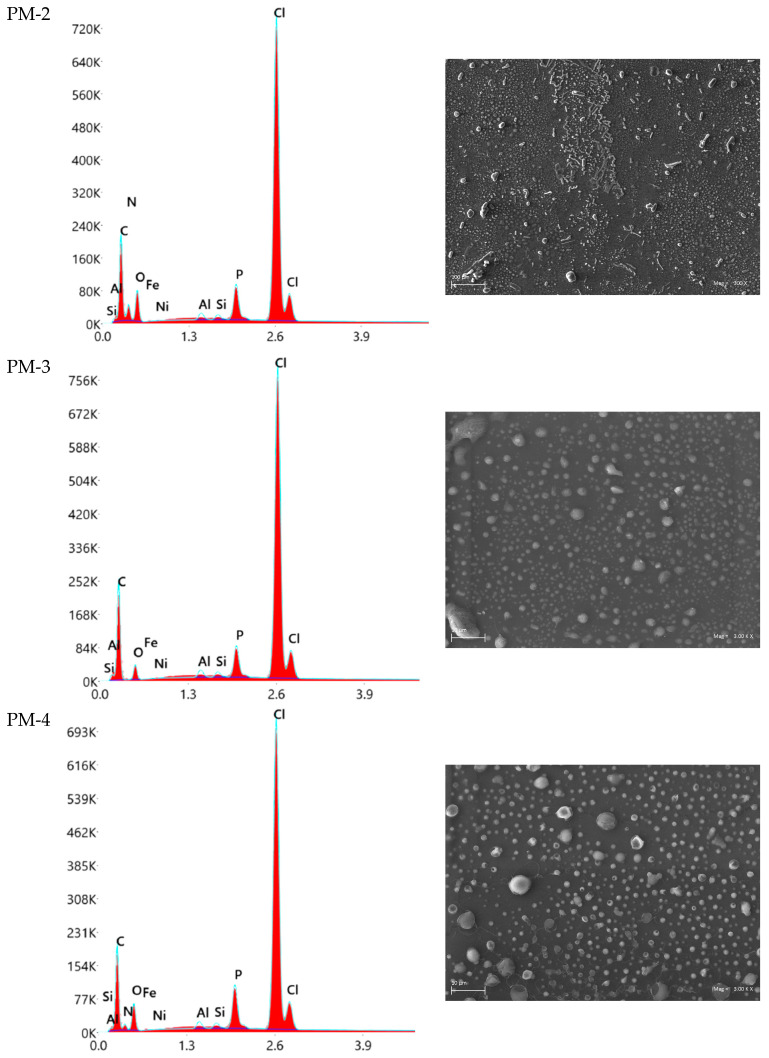

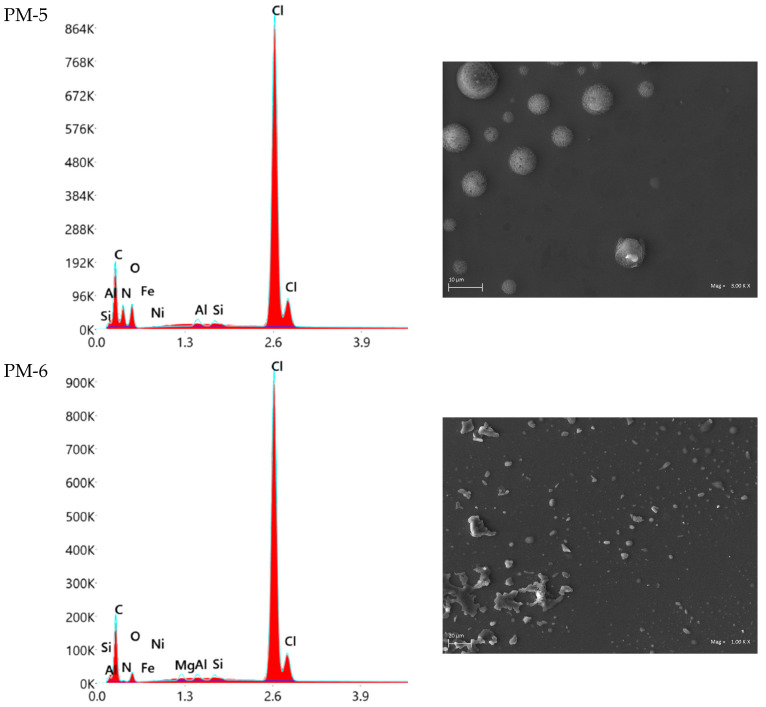
SEM-EDS images of PMs after metal ion sorption.

After the sorption processes, the SEM-EDS method was used to analyse and characterise the surface morphology and elemental composition of the tested PMs. It is known that additional polymer material components, e.g., chelating agent, can effect an increase or decrease in membrane voids [42,43]. The pure polymer material PM-0, which does not contain any chelating agent, showed a smooth and compact surface and did not exhibit any voids. In comparison, all tested PMs containing metal ion-binding agents, such as DESs (e.g., PM-1, PM-2, PM-5) or ILs (e.g., PM-3, PM-4, PM-6), demonstrated porous surfaces and voids. Thus, it was proven that the chelating agent, i.e., specific deep eutectic solvents or ionic liquids, were integrated with other components of the polymer materials, i.e., the polymer base and plasticiser. Figure 4 shows SEM-EDS images of all analysed PMs after metal ion sorption from alkaline battery leachate.

The SEM-EDS analysis also confirmed the presence of chelating agents in polymer materials. In the spectrogram of PM-0, only signals of C, O, and Cl derived from the polymer base and plasticiser are present (the weight (%) of the basic elements present in material: 76.9 (C), 7.2 (O), and 15.5 (Cl), respectively). In contrast, spectrograms of PM-3 and PM-4 additionally show signals of P originating from chelating agents Cyphos IL 101 and Cyphos IL 104 (the weight (%) of the basic elements present in materials: 78.4 (C), 5.3 (O), 14.7 (Cl), and 1.2 (P) in case of PM-3; 68.6 (C), 9.2 (O), 14.8 (Cl), 5.2 (N), and 1.6 (P) in case of PM-4). Likewise, spectrograms of PM-1 and PM-2 show signals related to used polymer base and plasticiser (C, O, Cl) and signals of P and N derived from deep eutectic solvents DES-1 and DES-2 (the weight (%) of the basic elements present in materials: 62.2 (C), 7.8(O), 17.0 (Cl), 11.4 (N), and 1.1 (P) in case of PM-1; 62.0 (C), 10.3 (O), 13.8 (Cl), 12.2 (N), and 1.3 (P) in case of PM-1). Lastly, spectrograms of PM-5 and PM-6 include signals of N originating from chelating agents DES-3 and Aliquat 336, respectively, as well as signals of C, O, and Cl from PVC and ADO (the weight (%) of the basic elements present in materials: 56.8 (C), 9.0(O), 16.0 (Cl), 17.7 (N) in case of PM-5; 75.1 (C), 4.2 (O), 18.4 (Cl), 1.6 (N) in case of PM-6). The analysis of polymer materials (PM-1 to PM-6) after sorption processes showed the presence of nickel(II) ions in addition to the basic elements of each membrane. The low weight percentage of nickel(II) ions is related to the nature of the initial solution (battery leachate) containing small amounts of Ni(II) ions.

### 3.5. Comparison of Metal Ion Separation Efficiency of ILs/DESs-Based PMs Using Literature Data

The systematic growth of interest in environmentally friendly methods for removing heavy metal ions from aqueous solutions (e.g., sewage, waste leachates) and other waste (e.g., electronic, ore processing waste) has led to numerous attempts to use various types of ILs or DESs as metal ion-binding agents in techniques designed for this purpose, such as solvent extraction, membrane separation, and adsorption [30,35,44]. However, despite the usually high efficiency of ionic liquids in such processes, their utilisation in some separation methods has recently become less common due to some drawbacks, such as the relatively high cost of their preparation and use (e.g., solvent extraction method uses relatively large amounts of ILs) and environmental concerns (ILs are usually not neutral to living organisms) [30]. Hence the increased interest in application of DESs in separation processes (e.g., membrane processes) of diverse nature. Moreover, deep eutectic solvents can be used both as metal ion carriers and instead of an aqueous solution as the permeate stream during electrodialysis [21]. Table 5 presents selected examples of the application of ILs (i.e., Cyphos IL 101, Cyphos IL 104, Aliquat 336) and various DESs in polymer materials intended for the separation of metal ions (e.g., Ni(II), Mn(II) and Zn(II)) from various solutions.

Ionic liquids such as Cyphos IL 101, Cyphos Il 104, or Aliquat 336 have been successfully used as metal ion-binding substances in various types of polymer materials (e.g., polymer inclusion membranes, polymer adsorptive membranes, polymer films) intended for the separation of different metal ions from various solutions (e.g., jewellery leachates, seawater, synthetic wastewater). However, the effectiveness of the separation of specific metal ions by specific polymer materials containing ILs depends on many factors, including the properties and amounts of other components in the material (e.g., the type and amount of polymer and plasticiser), the nature of the solution in which the separation is carried out (e.g., the amount and properties of ions present in the solution), and the conditions of the separation processes. Due to the number and complexity of factors influencing separation processes, it is not possible to develop one universal separation method for all metal ions, and it is necessary to determine the optimal parameters for different processes occurring in various solutions [45,46,47,48,49]. Recently, a polymer material containing DES (Aliquat 336 and diacetamide) was also successfully used for the separation of metal ions (Cu(II), Zn(II)) from computer scratch leachate. It was shown that although the separation efficiency of this DES-based material was comparable to that of the material containing Aliquat 336, the use of a deep eutectic solvent instead of an ionic liquid significantly shortened the time required for the removal of zinc(II) and copper(II) ions from analysed solution [28]. The results of the studies presented in this paper indicate that in the case of some DESs-based polymer materials (e.g., PM-1, PM-5), the efficiency of Ni(II) ion separation processes was slightly higher than in case of PMs containing corresponding ILs (PM-3, PM-6). However, in the case of separation of Mn(II) ions, the PM containing Cyphos IL 101 (PM-3) performed better than the one with Cyphos IL 101-based DES (PM-1). Although the differences in the efficiency of ILs-based and DESs-based PMs cannot be clearly determined based on these results, it can be assumed that due to the numerous advantages of the deep eutectic solvents, including their ability to bind various metal ions, further research will be conducted in this area.

**Table 5:** Selected examples of the use of ILs or DESs in polymer materials intended for the separation of metal ions.

Polymer Material Composition	Type of Solution	Removal Efficiency [%]	Main Advantages of Method	Ref.
Polymer inclusion membrane (PIM) consisting of cellulose triacetate (CTA) matrix, o-nitrophenyl pentyl ether (NPOE) plasticiser, and Cyphos IL 101	Model solutions of Cu(II), Zn(II) and Ni(II) ions and jewellery waste leachate	92% of Cu(II) 51% of Zn(II) >0.1% of Ni(II)	Ni(II) ions remained in the feed phase because they did not form anionic complexes with chloride ions.	[45]
PIM consisting of CTA, NPOE, and Cyphos IL 104		58% of Cu(II) 40% of Zn(II) >0.1% of Ni(II)		
Membranes comprising a mixture of CTA and poly(methyl methacrylate) (PMMA) dioctylephthalate (DOP) and phosphoric acid (D2EHPA)	Model solutions of Co(II), Cu(II), Ni(II), or Pb(II) ions	31.12% of Ni(II) 32.7% of Co(II) 17.1% of Cu(II) 21.85% of Pb(II)	Addition of Aliquat 336, D2EHPA, and TBP to the membranes enhances the wettability of the materials. A more hydrophilic membrane surface increases the efficiency of metal ion removal.	[47]
Membrane consisting of CTA, PMMA, DOP and Aliquat 336		100% of Ni(II) 23.5% of Co(II) 11.6% of Cu(II) 27.55% of Pb(II)		
Membrane consisting of CTA, PMMA, DOP, and tributyl phosphate (TBP)		4.48% of Ni(II) 29.5% of Co(II) 14.9% of Cu(II) 14.57% of Pb(II)		
Polymer membrane containing CTA, Aliquat 336 (or D2EHPA), and NPOE	Tecolutla seawater	87% of Pb(II) 90% of Cd(II) 94% of Zn(II)	As the concentration of metal ions decreased, the transport of metal ions increased (the competition for the active sites did not limit transport).	[48]
PIM composed of CTA, D2EHPA, and of acetylated kraft lignin (AKL)	Synthetic wastewater containing Cu(II) and Ni(II) ions	62% of Zn(II) 26% of Ni(II)	In case of binary Zn/Ni solution, a decrease in the extraction percentages of Zn(II) and Ni(II) ions was observed because the co-transport of Zn(II) with Ni(II) reduces the concentration of D2EHPA in the membrane.	[50]
Polymer adsorptive membranes (PAMs) based on PVC, ADO, and Aliquat 336	Acidic leachate of computer pins consisting of Zn(II) and Cu(II) ions	96.85% of Cu(II) 95.42% of Zn(II)	DES-containing PAM achieved approx. 97% of copper(II) and 95% of zinc(II) ions after 1 h of separation processes, while similar amounts of these metal ions were separated after 3 h with Aliquat 336-based PAM.	[28]
PAMs consisting of PVC, ADO, and DES (diacetamide-Aliquat 336)		96.63% of Cu(II) 95.42% of Zn(II)		

## 4. Conclusions

The results of application of different polymer materials containing, as metal ion-binding agents, ionic liquids (Cyphos IL 101 (PM-3), Cyphos IL 104 (PM-4), Aliquat 336 (PM-6)) or deep eutectic solvents composed of the corresponding ionic liquids and diacetamide (Cyphos IL 101/diacetamide (DES-1 in PM-1), Cyphos IL 104/diacetamide (DES-2 in PM-2), and Aliquat 336/diacetamide (DES-3 in PM-5)) for the separation of metal ions (Ni(II), Zn(II), and Mn(II)) from the acidic solution obtained by leaching black mass of alkaline batteries with aqua regia show that the efficiency of these processes differs significantly and strongly depends on both the composition of the PMs and the properties of the removed metal ions.

**Figure 5:**
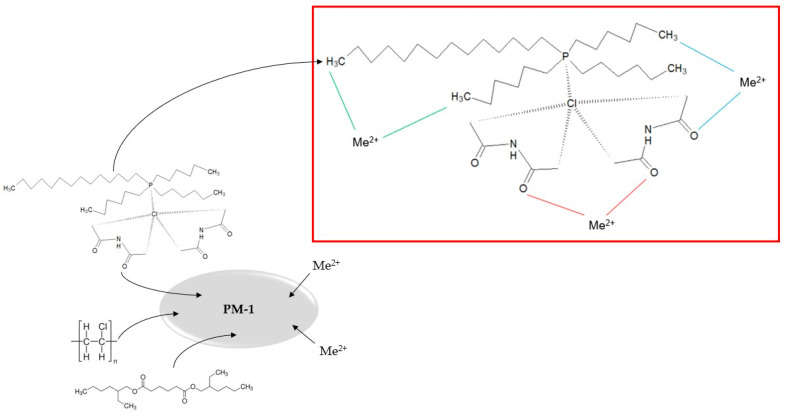
Simplified scheme of sorption of divalent metal ions (Me^2+^ = Ni^2+^, Mn^2+^, Zn^2+^) by PM containing DES-1. Colored lines indicate possible metal ion attachment sites.

All the synthesised PIMs were characterised by the highest efficiency in relation to the separation of nickel ions (%R_ads_ values ranged from about 58% (PM-2) to over 93% (PM-5)) and the lowest in the removal of manganese ions (the highest value of %R_ads_ = 14.28% was obtained for PM-3). In the case of the separation of zinc ions, the efficiency of all PMs was very similar and ranged from slightly over 35% (PM-4) to about 38% (PM-6). Replacing the ionic liquids Cyphos IL 101 or Aliquat 336 in polymer materials with corresponding deep eutectic solvents composed of the appropriate IL and diacetamide had a positive effect on the efficiency of nickel (II) ion separation. However, the use of PM containing Cyphos IL 104 enabled the recovery of nickel(II) ions with an efficiency of over 10% higher than in case of application of a material containing a Cyphos IL 104-based DES-2. Based on the results of performed experiments, it is not possible to clearly determine how metal ions bind to carriers (ILs or DESs) contained in synthesised polymer membranes during sorption processes. Figure 5 shows a simplified scheme of one of many possible mechanisms of such process.

The obtained results are promising in relation to the possibility of effective separation of metal ions from harsh acidic solutions, using novel, easy-to-synthesise polymer materials containing deep eutectic solvents (especially DES-1 and DES-5). It should be emphasised that the processes of synthesis of DESs itself (i.e., mixing the components in appropriate proportions and heating) are also relatively easy, simple, and inexpensive. In addition, the obtained results of the PMs contact angle measurement show that they are hydrophilic, which is beneficial in terms of reducing material surface contamination and should generally contribute to increased material efficiency [28]. However, further research is necessary, for example, related to the analysis of the properties of DESs containing ILs and diacetamide in slightly different proportions, to the influence of such DESs on the properties of PMs, or to the possibility of multiple use of such novel polymer materials. The development of effective methods of PM regeneration, enabling the reuse and proper management of PMs after exhaustion of their separation capacity, is important not only in the context of reducing the costs of separation processes, but also when considering that they may also have an impact on the environment (reduction in chemical consumption, recycling of materials). It is also important to undertake research to determine the optimal conditions for the separation processes (e.g., determining the process time, the effect of pH, the content of competing ions, etc.). In the case of separation processes designed to be carried out on a larger scale, it is also necessary to estimate the process costs and their profitability. Potentially, polymer materials containing DESs, which are considered as environmentally safe substances, may also be used in the future for wastewater treatment, e.g., for the removal of metal ions from acidic industrial wastewater on a larger scale.

## Figures and Tables

**Table 1:** Composition of the prepared DESs (with HBD:HBA molar ratio of 2:1) and selected physicochemical properties of their constituents [29].

HBD						
Symbol of DES	Name	Mass [g]	Molar Mass [g/mol]	Density [g/mL]	Melting Point [°C]	Boiling Point [°C]
DES-1	Diacetamide	0.385	101.1	1.3846	75.5–76	222–233
DES-2	Diacetamide	0.265				
DES-3	Diacetamide	0.501				
**HBA**						
**Symbol of DES**	**Name**	**Mass [g]**	**Molar Mass [g/mol]**	**Density [g/mL]**	**Melting Point [°C]**	**Boiling Point [°C]**
DES-1	Cyphos IL 101	1.009	519.31	0.895	−70	no data *
DES-2	Cyphos IL 104	1.016	773.27	0.887	−70	no data *
DES-3	Aliquat 336	1.015	446.25	0.88	−20	225

* No single, standard boiling point.

**Table 2:** Composition of obtained PMs.

Symbol of PM	Polymer Base		Chelating Agent		Plasticiser	
	Name	Mass [g]	Name	Mass [g]	Name	Mass [g]
PM-0	PVC	0.504	-	-	ADO	0.236
PM-1	PVC	0.509	DES-1	0.215	ADO	0.203
PM-2	PVC	0.505	DES-2	0.208	ADO	0.214
PM-3	PVC	0.505	Cyphos IL 101	0.196	ADO	0.194
PM-4	PVC	0.503	Cyphos IL 104	0.201	ADO	0.200
PM-5	PVC	0.504	DES-3	0.203	ADO	0.214
PM-6	PVC	0.508	Aliquat 336	0.205	ADO	0.204

Weights of components before mixing.

**Table 3:** Contact angles of synthesised polymer materials.

CA	74.42°	78.9°	60.32°	69.52°	43.8°
Figure	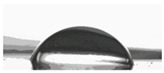	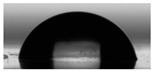	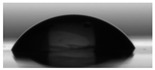	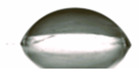	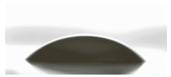
PM	PM-0	PM-1	PM-2	PM-3	PM-4

**Table 4:** Sorption capacities of investigated polymer materials after a 1 h sorption process in an aqua regia leachate of alkaline battery black mass. The average q_t_ in all experiments was ±0.000001.

PMs	q_t_ [mg/g]		
	Ni(II)	Mn(II)	Zn(II)
PM-0	0.000034	0.000676	0.077703
PM-1	0.000676	0.002365	0.556983
PM-2	0.000719	0.000676	0.529591
PM-3	0.000763	0.002027	0.548274
PM-4	0.000553	0.001014	0.548274
PM-5	0.000766	0.001014	0.566521
PM-6	0.000888	0.001689	0.553203
PM-0	0.000034	0.000676	0.077703

## Data Availability

The original contributions presented in this study are included in the article/Supplementary Material. Further inquiries can be directed to the corresponding author.

## Supplementary Materials

The following supporting information can be downloaded at: https://www.mdpi.com/article/10.3390/ma18122768/s1, Figure S1A: (a) The 1H NMR spectrum of DES-1, (b) enlarged fragment of the 1H NMR spectrum of DES-1; Figure S1B: (a) The 13C NMR spectrum of DES-1, (b) enlarged fragment of the 13C NMR spectrum of DES-1; Figure S2A: (a) The 1H NMR spectrum of DES-2, (b) enlarged fragment of the 1H NMR spectrum of DES-2; Figure S2B: (a) The 13C NMR spectrum of DES-2, (b) enlarged fragment of the 13C NMR spectrum of DES-2; Figure S3: The 1H NMR spectra of pure diacetamide (a), Cyphos IL 101 (b), and Cyphos IL 104 (c); Figure S4: The FTIR-ATR spectrum of polymer material PM-0, containing polymer PVC and plasticiser ADO; Figure S5: The FTIR-ATR spectrum of polymer material PM-1, containing polymer PVC, plasticiser ADO and DES-1 (composed of Cyphos IL 101 and diacetamide); Figure S6: The FTIR-ATR spectrum of polymer material PM-2, containing polymer PVC, plasticiser ADO and DES-2 (composed of Cyphos IL 104 and diacetamide); Figure S7: The FTIR-ATR spectrum of polymer material PM-5, containing polymer PVC, plasticiser ADO and DES-3 (composed of Aliquat 336 and diacetamide); Figure S8: The FTIR-ATR spectrum of polymer material PM-6, containing polymer PVC, plasticiser ADO and ionic liquid Aliquat 336.

## References

[1] Hu X., Robles A., Vikström T., Väänänen P., Zackrisson M., Ye G. (2021). A novel process on the recovery of zinc and manganese from spent alkaline and zinc-carbon batteries. J. Hazard. Mater..

[2] Gianvincenzi M., Mosconi E.M., Marconi M., Tola F. (2024). Battery waste management in Europe: Black mass hazardousness and recycling strategies in the light of an evolving competitive regulation. Recycling.

[3] Dar A.A., Chen Z., Zhang G., Hu J., Zaghib K., Deng S., Wang X., Haghighat F., Mulligan C.N., An C. (2025). Sustainable Extraction of Critical Minerals from Waste Batteries: A Green Solvent Approach in Resource Recovery. Batteries.

[4] Regulation (EU) 2023/1542 of the European Parliament and of the Council Concerning Batteries and Waste Batteries Amending Directive 2008/98/EC and Regulation (EU) 2019/1020 and Repealing Directive 2006/66/EC. https://eur-lex.europa.eu/eli/reg/2023/1542/oj/eng.

[5] Ginster R., Blömeke S., Popien J.-L., Scheller C., Cerdas F., Herrmann C., Spengler T.S. (2024). Circular battery production in the EU: Insights from integrating life cycle assessment into system dynamics modeling on recycled content and environmental impacts. J. Ind. Ecol..

[6] Cui K., Zhao M.-C., Li Y., Atrens A., Zhang F. (2025). Recycling of spent lithium iron phosphate batteries: Research progress based on environmental protection and sustainable development technology. Sep. Purif. Technol..

[7] Tran L.-H., Tanong K., Jabir A.D., Mercier G., Blais J.-F. (2020). Hydrometallurgical process and economic evaluation for recovery of zinc and manganese from spent alkaline batteries. Metals.

[8] Dehghani-Sanij A.R., Tharumalingam E., Dusseault M.B., Fraser R. (2019). Study of energy storage systems and environmental challenges of batteries. Renew. Sustain. Energy Rev..

[9] Valdrez I.V., Almeida M.F., Dias J.M. (2022). Direct recovery of Zn from wasted alkaline batteries through selective anode’s separation. J. Environ. Manag..

[10] Skrzekut T., Piotrowicz A., Noga P., Wędrychowicz M., Bydałek A.W. (2022). Studies of selective recovery of zinc and manganese from alkaline batteries scrap by leaching and precipitation. Materials.

[11] Baba A.A., Adekola F.A., Bale R.B., Alabi A.G.F., Raji M.A., Chen X., Zhong Y., Zhang L., Howarter J.A., Baba A.A., Wang C., Sun Z., Zhang M., Olivetti E., Luo A. (2020). Economic metals rescue from spent zinc–carbon batteries for industrial value additions. Energy Technology 2020: Recycling, Carbon Dioxide Management, and Other Technologies.

[12] Sobianowska-Turek A., Szczepaniak W., Maciejewski P., Gawlik-Kobylińska M. (2016). Recovery of zinc and manganese, and other metals (Fe, Cu, Ni, Co, Cd, Cr, Na, K) from Zn-MnO_2_ and Zn-C waste batteries: Hydroxyl and carbonate co-precipitation from solution after reducing acidic leaching with use of oxalic acid. J. Power Sources.

[13] García N.M., Cano B.D., Valverde J.L., Heitz M., Ramirez A.A. (2024). Extraction and separation of potassium, zinc and manganese issued from spent alkaline batteries by a three-unit hydrometallurgical process. J. Chem. Technol. Biotechnol..

[14] Lannoo S., Vilas-Boas A., Maryam Sadeghi S., Jesus J., Soares H.M.V.M. (2019). An environmentally friendly closed loop process to recycle raw materials from spent alkaline batteries. J. Clean. Prod..

[15] Maryam Sadeghi S., Jesus J., Soares H.M.V.M. (2020). A critical updated review of the hydrometallurgical routes for recycling zinc and manganese from spent zinc-based batteries. Waste Manag..

[16] Swain B. (2017). Recovery and recycling of lithium: A review. Sep. Purif. Technol..

[17] Domańska U., Wiśniewska A., Dąbrowski Z., Kolasa D., Wróbel K., Lach J. (2024). Recovery of Metals from the “Black Mass” of Waste Portable Li-Ion Batteries with Choline Chloride-Based Deep Eutectic Solvents and Bi-Functional Ionic Liquids by Solvent Extraction. Molecules.

[18] Elhamarnah Y., Qiblawey H., Nasser M. (2024). A review on deep eutectic solvents as the emerging class of green solvents for membrane fabrication and separations. J. Mol. Liq..

[19] Taghizadeh M., Taghizadeh A., Vatanpour V., Ganjali M.R., Saeb M.R. (2021). Deep eutectic solvents in membrane science and technology: Fundamental preparation, application, and future perspective. Sep. Purif. Technol..

[20] Jha D., Maheshwari P., Singh Y., Haider M.B., Kumar R., Balathanigaimani M.S. (2023). A comparative review of extractive desulfurization using designer solvents: Ionic liquids & deep eutectic solvents. J. Energy Inst..

[21] Siekierka A., Callahan D.L., Kujawski W., Dumée L.F. (2024). Deep eutectic solvent assisted electrodialysis towards selective resource recovery from model spent batteries effluents. Desalination.

[22] Abranches D.O., Coutinho J.A.P. (2023). Everything you wanted to know about deep eutectic solvents but were afraid to be told. Annu. Rev. Chem. Biomol. Eng..

[23] Duque A., Sanjuan A., Bou-Ali M.M., Alonso R.S., Campanero M.A. (2023). Physicochemical characterization of hydrophobic type III and type V deep eutectic solvents based on carboxylic acids. J. Mol. Liq..

[24] Liu L., Zhu G., Huang Q., Yin C., Jiang X., Yang X., Xie Q. (2021). Efficient recovery of Au(III) through PVDF-based polymer inclusion membranes containing hydrophobic deep eutectic solvent. J. Mol. Liq..

[25] Bastos H., Gallestegui A., de Lacalle J.L., Schaeffer N., Pringle J.M., Mecerreyes D., Pozo-Gonzalo C. (2024). Ionic polymer absorbents inspired by deep eutectic solvents to recover cobalt and nickel. New J. Chem..

[26] Carreira A.R.F., Nogueira A., Crema A.P.S., Passos H., Schaeffer N., Coutinho J.A.P. (2023). Super concentrated HCl in a deep eutectic solvent as media for the integrated leaching and separation of metals from end-of-life lithium-ion batteries. J. Chem. Eng..

[27] Zhang Y., Cui P., Luo G., Chen L., Li X., Chao Y., Zhu W. (2023). One-step selective separation and efficient recovery of valuable metals from spent lithium batteries by phosphoric acid-base deep eutectic solvent. Green Chem. Eng..

[28] Kaczorowska M.A., Bożejewicz D., Witt K. (2023). Application of deep eutectic mixture and ionic liquid as carrier in polymer adsorptive membranes for removal of copper(II) and zinc(II) ions from computer scrap leachates. Desalin. Water Treat..

[29] https://www.chemicalbook.com/.

[30] Khazalpour S., Yarie M., Kianpour E., Amani A., Asadabadi S., Seyf J.-Y., Razaeivala M., Azizian S., Zolfigol M.A. (2020). Applications of phosphonium-based ionic liquids in chemical processes. J. Iran Chem. Soc..

[31] Liu C., Mei G., Yu M., Cheng Q., Yang S. (2021). New applications of deep eutectic solvents for separation of quartz and magnetite. Chem. Phys. Lett..

[32] Sant’Ana P.L., Bortoleto J.R.R., da Cruz N.C., Rangel E.C., Durrant S.F., Schreiner W.H. (2020). Surface functionalization of polyvinyl chloride by plasma immersion techniques. Polímeros.

[33] Govindappa H., Bhat M.P., Uthappa U.T., Sriram G., Altalhi T., Kumar S.P., Kurkuri M. (2022). Fabrication of novel polymer inclusion membrane from recycled polyvinyl chloride for the real-time extraction of arsenic(V) from water samples in a continuous process. Chem. Eng. Res. Des..

[34] Jiang H.-L., Lin J.-C., Hai W., Tan H.-W., Luo Y.-W., Xie X.-L., Cao Y., He F.-A. (2019). A novel crosslinked β-cyclodextrin-based polymer for removing methylene blue from water with high efficiency. Colloids Surf. A Physicochem. Eng. Asp..

[35] Witt K., Kaczorowska M.A., Bożejewicz D. (2024). Efficient, fast, simple, and eco-friendly methods for separation of toxic chromium(VI) ions based on ion exchangers and polymer materials impregnated with Cyphos IL 101, Cyphos IL 104, or D2EHPA. Environ. Sci. Pollut. Res..

[36] https://chem.libretexts.org/Ancillary_Materials/Reference/Reference_Tables/Spectroscopic_Reference_Tables/Infrared_Spectroscopy_Absorption_Table.

[37] Bożejewicz D., Witt K., Kaczorowska M.A. (2023). Influence of the type of polymer and plasticizer on the properties and efficiency of membranes containing acetylacetone carrier for the removal of Cd(II) ions from aqueous solution. Desalin. Water Treat..

[38] Xu H., Xu D.C., Wang Y. (2017). Natural indicates for the chemical hardness/softness of metal cations and ligands. ACS Omega.

[39] Witt K., Bożejewicz D., Kaczorowska M.A. (2020). *N,N′*-Bis(salicylidene)ethylenediamine (Salen) as an active compound for the recovery of Ni(II), Cu(II), and Zn(II) ions from aqueous solution. Membranes.

[40] Gupta S.S., Bhattacharyya K.G. (2014). Adsorption of metal ions by clays and inorganic solids. RSC Adv..

[41] Admeliluyi F.T., Nze J.C. (2016). Multiple adsorption of heavy metal ions in aqueous solution using activated carbon from Nigerian bamboo. Int. J. Res. Eng. Technol..

[42] Tarhouchi S., Hlaibi M. (2024). Kinetic control aspects and mechanisms in oriented membrane process for extraction and recovery of ascorbic acid compound. Environ. Sci. Pollut. Res..

[43] Ghasemi H., Abu-Zahra N., Baig N., Abdulazeez I., Aljundi I.H. (2023). Enhancing fouling resistance and separation performance of polyethersulfone membrane through surface grafting with copolymerized termo-responsive polymer and copper oxide nanoparticles. Chem. Eng. J. Adv..

[44] Majidi E., Bakhshi H. (2024). Hydrophobic deep eutectic solvents characterization and performance for efficient removal of heavy metals from aqueous media. J. Water Process Eng..

[45] Radzymińska-Lenarcik E., Pyszka I., Urbaniak W. (2023). The use of polymer membranes for the recovery of copper, zinc and nickel from model solutions and jewellery waste. Polymers.

[46] Kagaya S., Hida-Matsuda K., Tsuzaka S., Minami C., Gemmei-Ide M., Cattrall R.W., Kolev S.D. (2023). The determination of zinc using flow injection and continuous flow analysis combined with a polymer inclusion film-coated column: Application to the determination of zinc in alloys and commercial lithium chloride. Talanta.

[47] Mesrouk S., Sadi F. (2024). Synthesis and characterization of low-cost plasticized polymeric membranes for separation of bivalent cations. Cellulose Chem. Technol..

[48] Macías M., de San Miguel R.E. (2023). On the Use of Polymer Inclusion Membranes for the Selective Separation of Pb(II), Cd(II), and Zn(II) from Seawater. Membranes.

[49] Ncib S., Chibani A., Barhoumi A., Larchet C., Dammak L., Elaloui E., Bouguerra W. (2023). Separation of copper and nickel from synthetic wastewater by polymer inclusion membrane containing di(2-ethylhexyl)phosphoric acid. Polym. Bull..

[50] Mahmoud H., Ncib S., Othmen K., Al-Hazmy S.M., Dammak L., Elaloui E., Bouguerra W. (2024). Evaluation of poly(vinyl chloride)/2-nitrophenyl octyl ether/di(2-ethylhexyl) phosphoric acid polymer inclusion membrane performance for zinc recovery and separation. Chem. Afr..

