# Supplementary material for: Application of Novel Polymer Materials Containing Deep Eutectic Solvents for the Separation of Metal Ions from Alkaline Battery Leachates
Source: Materials (Basel). 2025 Jun 12;18(12):2768. doi: 10.3390/ma18122768 (PMC12195004; doi:10.3390/ma18122768)
Supplement: Supplementary file 1 [file materials-18-02768-s001.zip › materials-3642077-supplementary.pdf]

materials

MDPI

Supplementary materials to the manuscript:

Application of Novel Polymer Materials Containing Deep Eutectic Solvents for the Separation of Metal Ions from Alkaline Battery Leachates

Daria Bożejewicz and Małgorzata A. Kaczorowska *

Faculty of Chemical Technology and Engineering, Bydgoszcz University of Science and Technology, Seminaryjna 3 Street, 85-326 Bydgoszcz, Poland
* Correspondence: malgorzata.kaczorowska@pbs.edu.pl

The figures below (S1A, S1B, S2A, S2B) present the ^{1}H NMR and ^{13}C NMR spectra of DES-1 and DES-2, respectively, obtained using a Bruker Avance III 400 MHz spectrometer (with DMSO-d6 solution).

a)

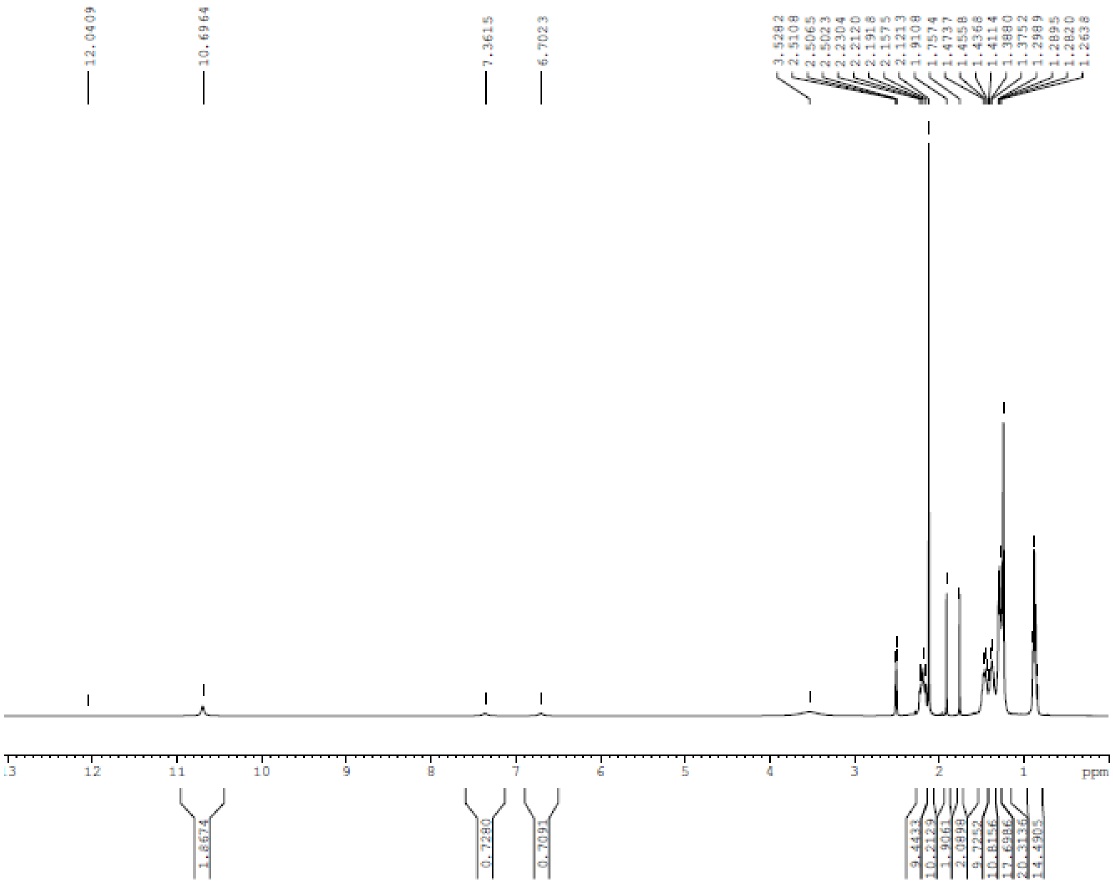

b)

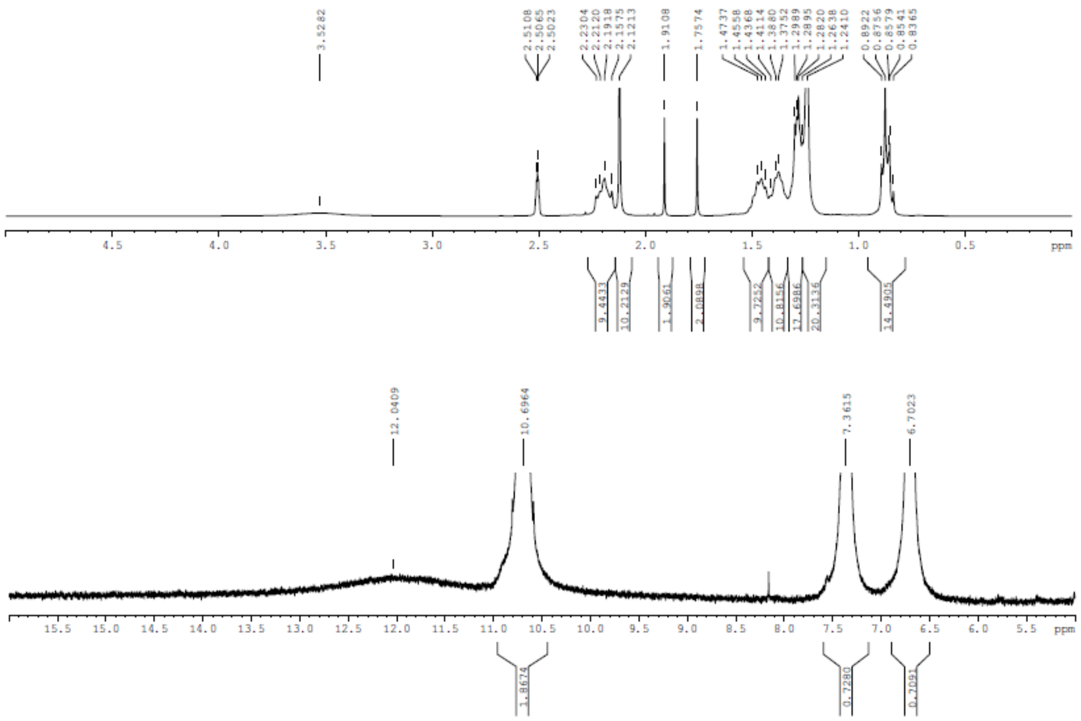

Figure S1. A. a) The ^{1}H NMR spectrum of DES-1, b) enlarged fragment of the ^{1}H NMR spectrum of DES-1. Main spectral data of DES-1, ^{1}H NMR: 400 Hz, DMSO, δ 10.6964 (1H, s), 7.3625 (1H, s), 6.7023 (1H, s), 2.2304-2.1575 (7H, m), 2.1213 (8H, s), 1.9108 (1H, s), 1.7574 (2H, s), 1.4737-1.4368 (7H, J=7.3 Hz, t), 1.4114-1.3752 (8H, J=7.3Hz, t), 1.2989-1.2820 (13, m), 1.2638-1.2410 (15, d), 0.8922-0.8365 (11H, m).

a)

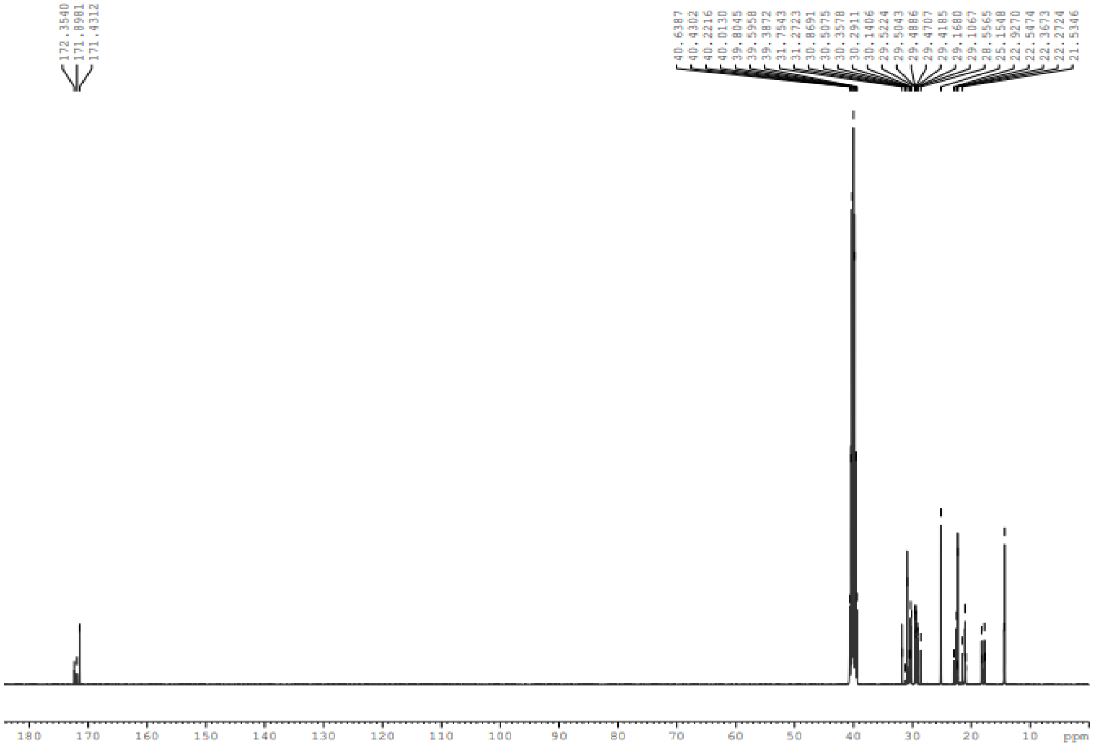

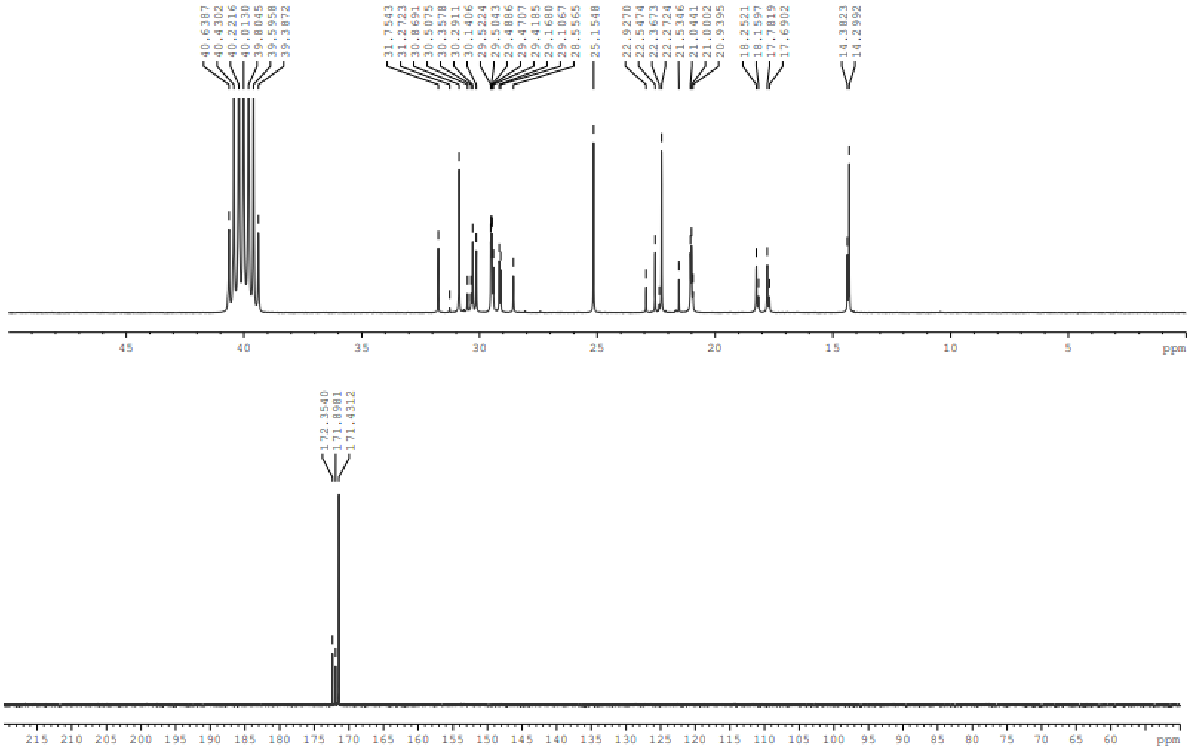

Figure S1. B. a) The ^{13}C NMR spectrum of DES-1, b) enlarged fragment of the ^{13}C NMR spectrum of DES-1. Main spectral data of DES-1, ^{13}C NMR: 101 Hz, DMSO, δ 172.3540, 171.8981, 171.4312, 40.6387-39.3872, 31.7543, 31.2723, 30.8691, 30.5075, 30.3578, 30.2911, 30.1406, 29.5224, 29.5043, 29.4886, 29.4707, 29.4185, 29.1680, 29.1067, 28.5565, 25.1548, 22.9270, 22.5474, 22.3673, 22.2724, 21.5346, 21.0441, 21.0002, 20.9395, 18.2521, 18.1597, 17.7819, 17.6902, 14.3823, 14.2992. The signal of the P magnetic nucleus appeared as a multiplet.

a)

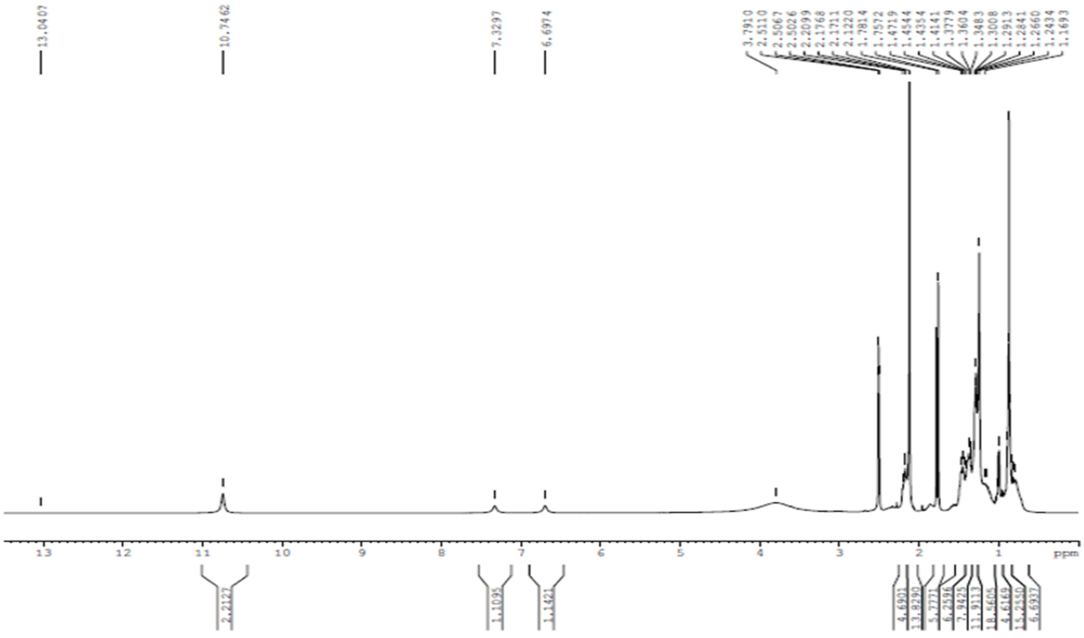

b)

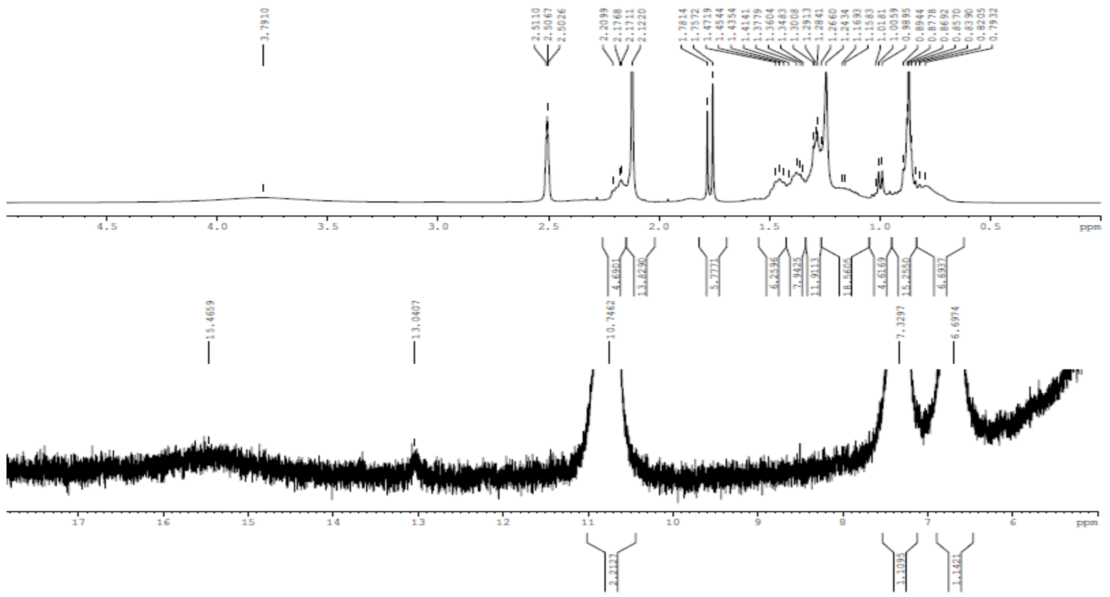

Figure S2. A. a) The ^{1}H NMR spectrum of DES-2, b) enlarged fragment of the ^{1}H NMR spectrum of DES-2. Main spectral data of DES-1, ^{1}H NMR: 400 Hz, DMSO, δ 10.7462 (2H, s), 7.3297 (1H, s), 6.6974 (1H, s), 2.2099-2.1711 (5H, m), 2.1220 (15H, s), 1.7814-1.7572 (6H, d), 1.4719-1.4141 (6H, m), 1.3779-1.3483 (9H, m), 1.3008-1.2841 (13H, m), 1.2660-1.1583 (20H, m), 1.0181-0.9895 (17H, m), 0.8692-0.7932 (7H, t).

a)

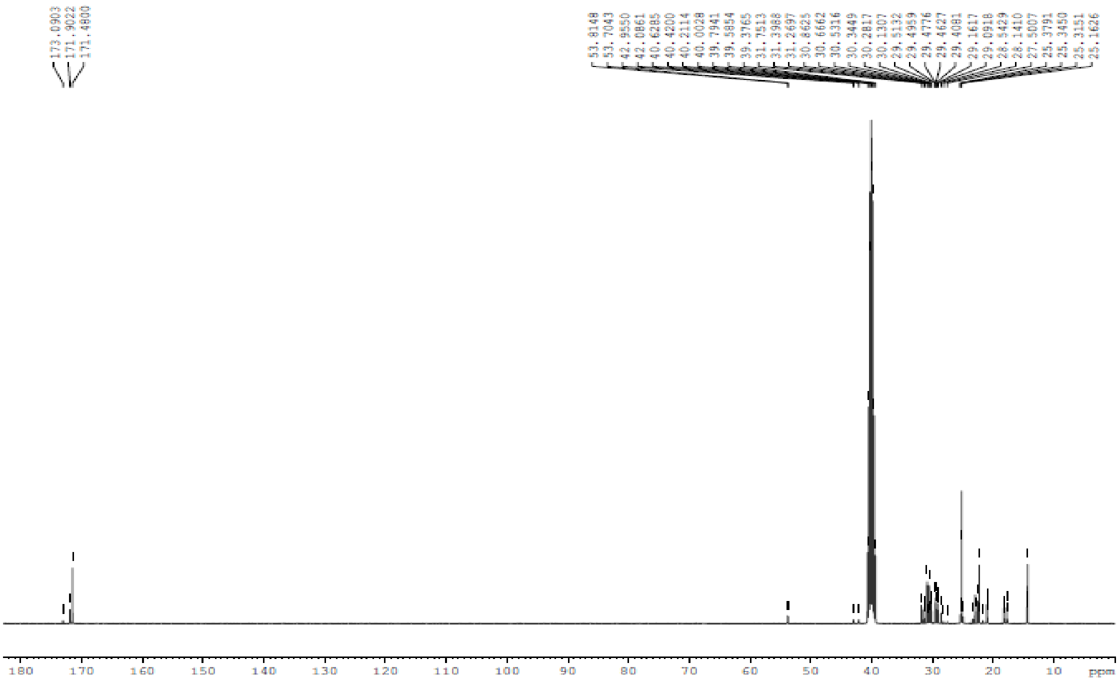

b)

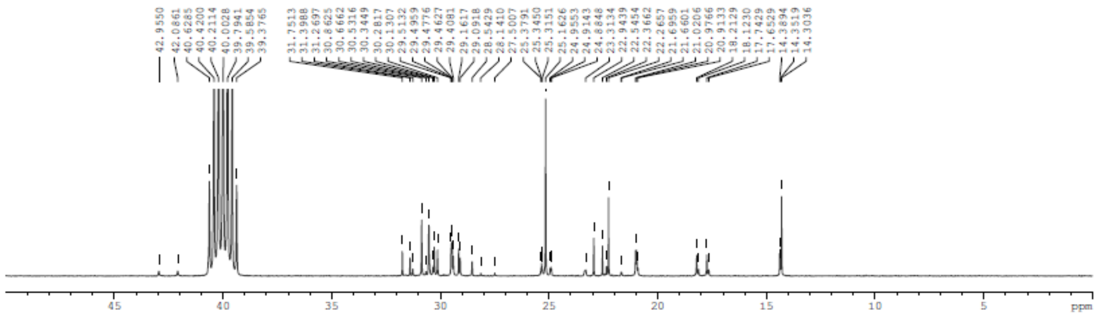

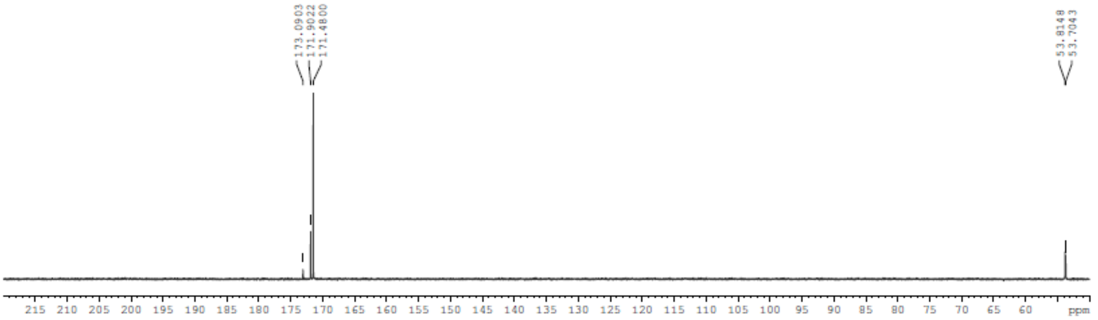

Figure S2. B. a) The ^{13}C NMR spectrum of DES-2, b) enlarged fragment of the ^{13}C NMR spectrum of DES-2. Main spectral data of DES-2, ^{13}C NMR: 101 Hz, DMSO, δ173.0903-171.4800, 53.8148-53.7043, 42.9550, 42.0861, 40.6285-39.3765, 31.7513, 31.3988, 31.2697, 30.8625, 30.6662-30.1307, 29.5132-29.4081, 29.1617-29.0918, 28.5429, 28.1410, 27.5007, 25.3791-25.3151, 25.1626, 24.9553-24.8848, 23.3134, 22.9439, 22.5454, 22.3662, 22.2657, 21.6959, 21.6601, 21.0206-20.9133, 18.2129-18.1230, 17.7429-17.6529, 14.3894-14.036. The signal of the P magnetic nucleus appeared as a multiplet.

a)

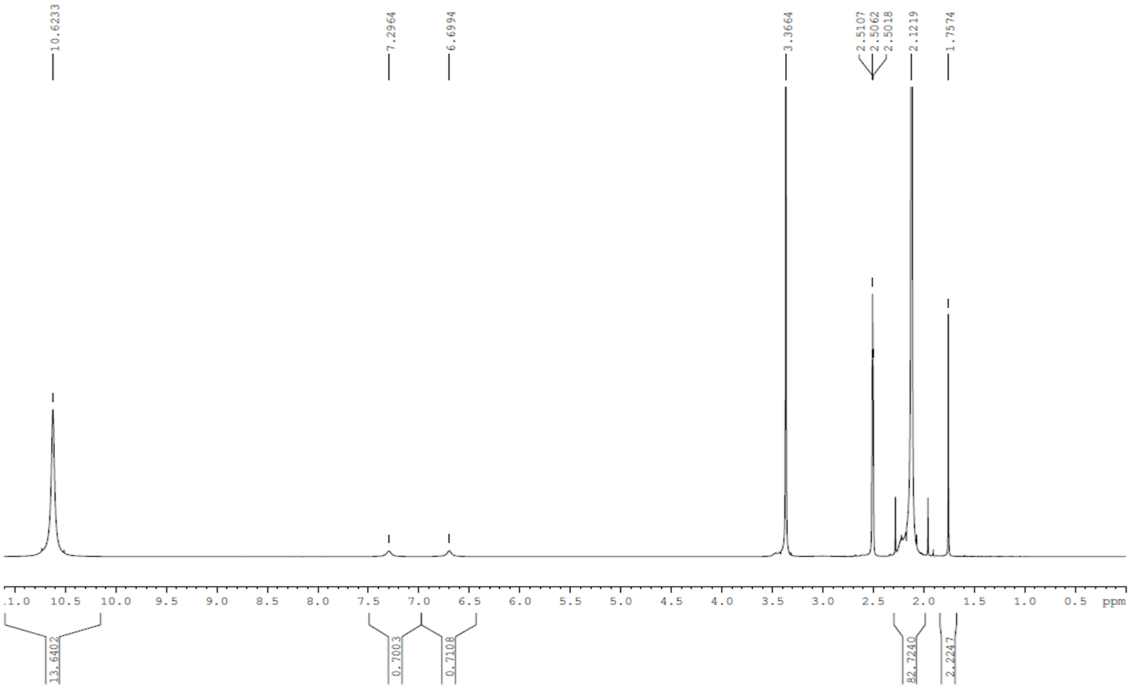

b)

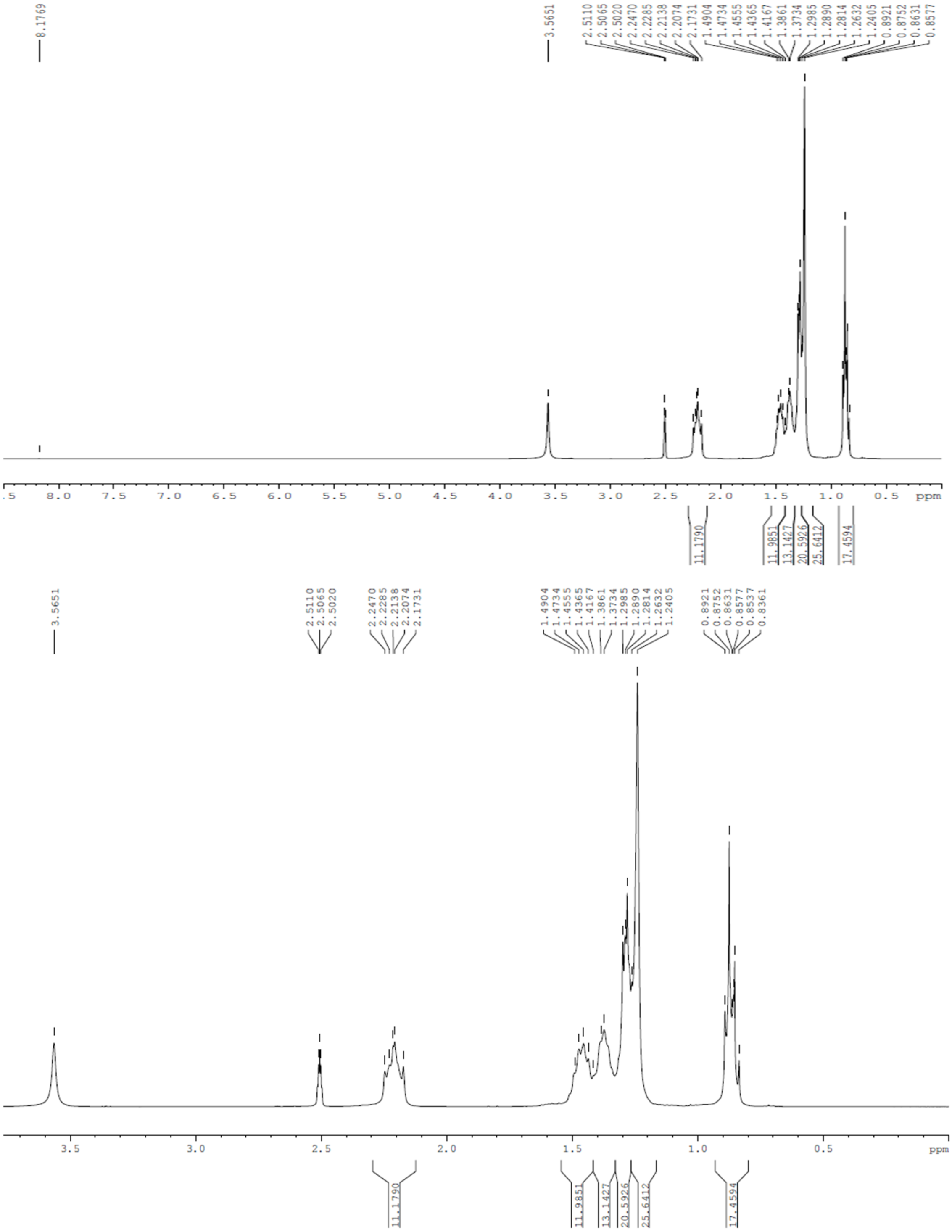
8.1769
3.5651
2.5110 2.5065 2.5020 2.2470 2.2285 2.2138 2.2074 2.1731 1.4904 1.4734 1.4555 1.4365 1.4167 1.3861 1.3734 1.2985 1.2890 1.2814 1.2632 1.2405 0.8921 0.8752 0.8631 0.8577
8.0 7.5 7.0 6.5 6.0 5.5 5.0 4.5 4.0 3.5 3.0 2.5 2.0 1.5 1.0 0.5 ppm
11.1790 11.9851 13.1427 20.5926 25.6412 17.4594
3.5651
2.5110 2.5065 2.5020
2.2470 2.2285 2.2138 2.2074 2.1731
1.4904 1.4734 1.4555 1.4365 1.4167 1.3861 1.3734 1.2985 1.2890 1.2814 1.2632 1.2405
0.8921 0.8752 0.8631 0.8577 0.8537 0.8361
3.5 3.0 2.5 2.0 1.5 1.0 0.5 ppm
11.1790 11.9851 13.1427 20.5926 25.6412 17.4594

c)

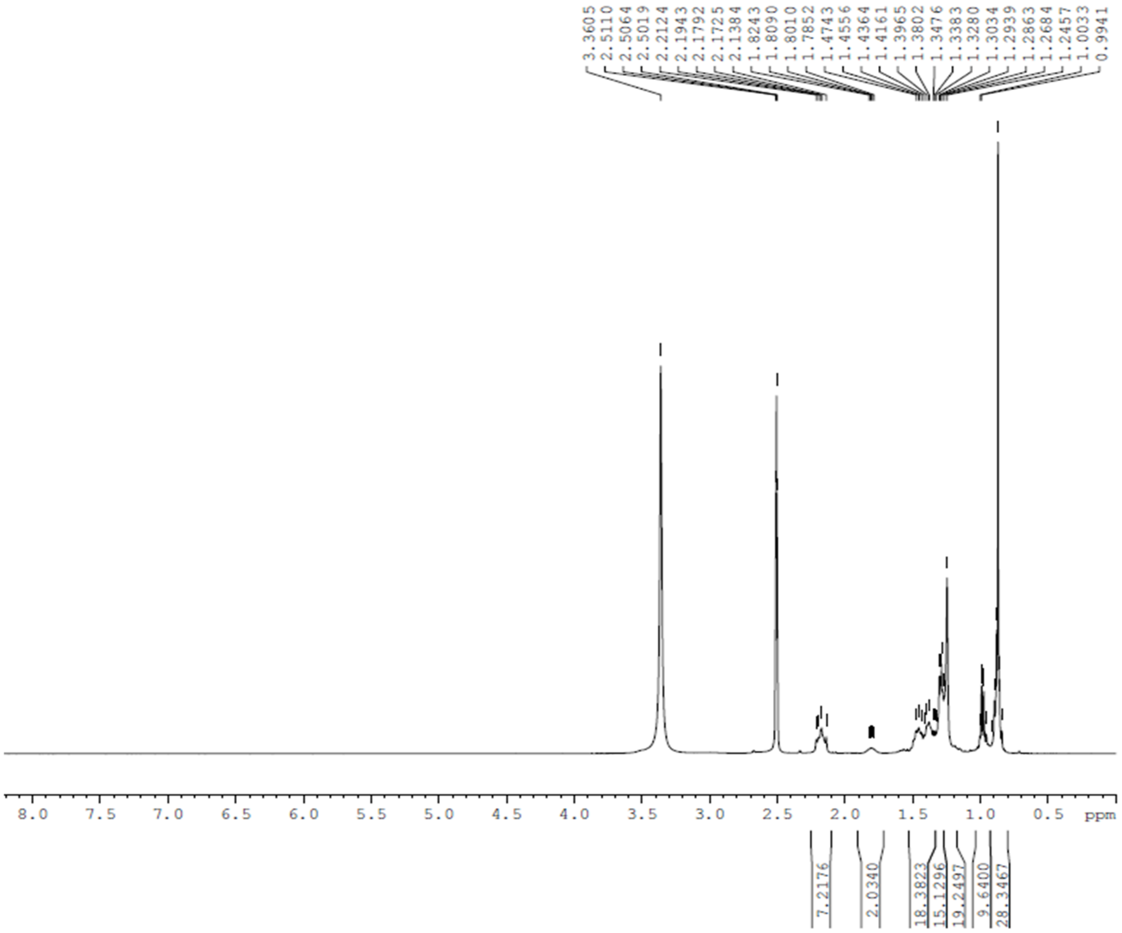
3.3605
2.5110
2.5064
2.5019
2.2124
2.1943
2.1792
2.1725
2.1384
1.8243
1.8090
1.8010
1.7852
1.4743
1.4556
1.4364
1.4161
1.3965
1.3802
1.3476
1.3383
1.3280
1.3034
1.2939
1.2863
1.2684
1.2457
1.0033
0.9941
8.0 7.5 7.0 6.5 6.0 5.5 5.0 4.5 4.0 3.5 3.0 2.5 2.0 1.5 1.0 0.5 ppm
7.2176
2.0340
18.3823
15.1296
19.2497
9.6400
28.3467

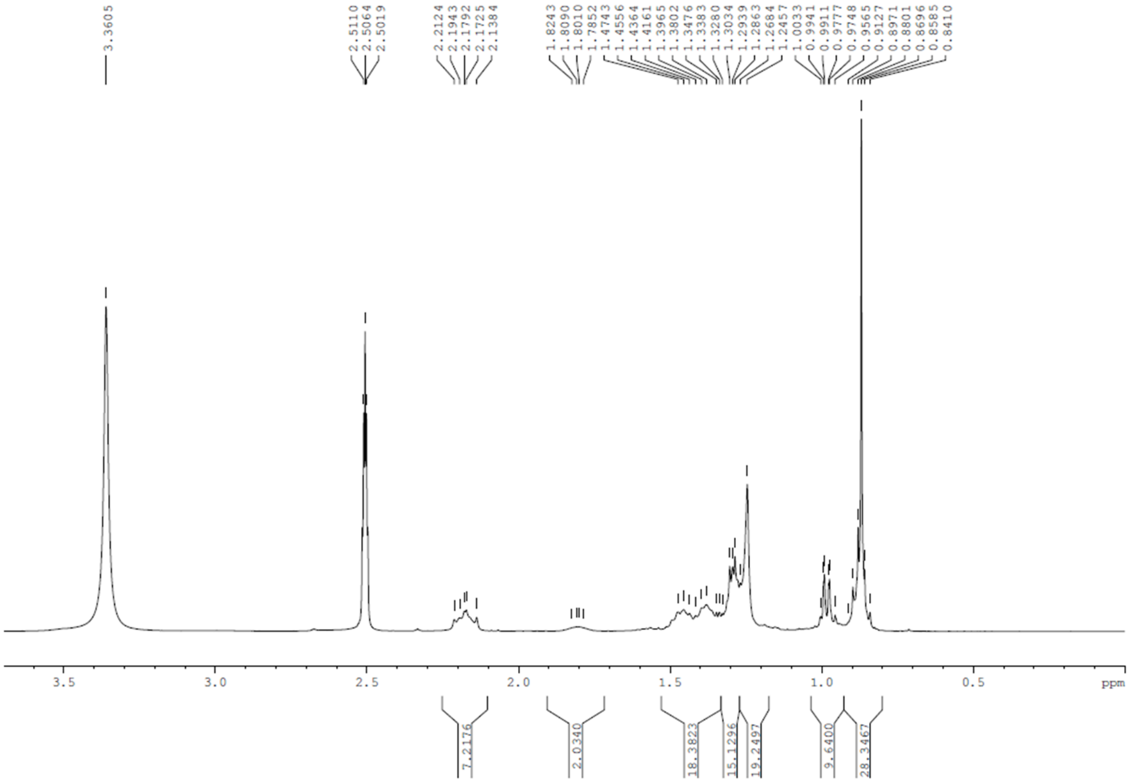
3.3605
2.5110
2.5064
2.5019
2.2124
2.1943
2.1792
2.1725
2.1384
1.8243
1.8090
1.8010
1.7852
1.4743
1.4556
1.4364
1.4161
1.3965
1.3802
1.3476
1.3383
1.3280
1.3034
1.2939
1.2863
1.2684
1.2457
1.0033
0.9941
0.9911
0.9777
0.9748
0.9565
0.9127
0.8971
0.8801
0.8696
0.8585
0.8410
3.5 3.0 2.5 2.0 1.5 1.0 0.5 ppm
7.2176
2.0340
18.3823
15.1296
19.2497
9.6400
28.3467

Figure S3. The ^{1}H NMR spectrum of pure diacetamide (a), Cyphos IL 101 (b), and Cyphos IL 104 (c). In the case of Ils ^{1}H NMR spectra (b and c), their enlarged fragments are also provided below the main spectra.

Figures S4–S8 present the FTIR-ATR spectra of the investigated polymer materials: PM-0, PM-1, PM-2, PM-5 and PM-6

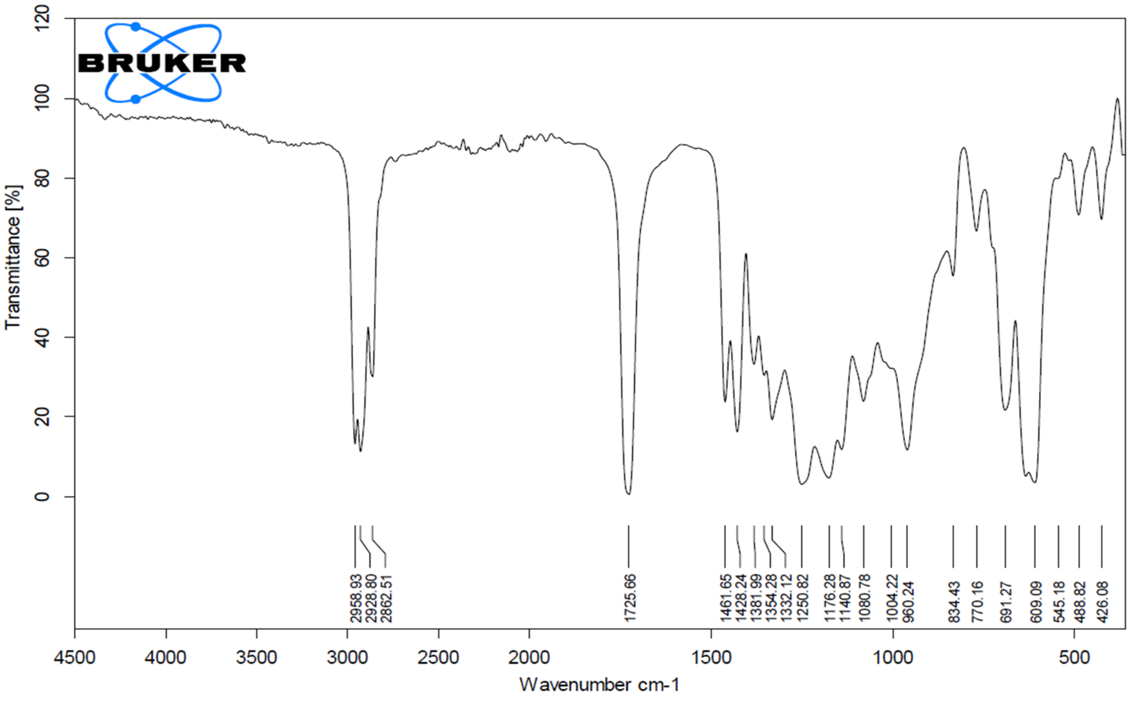

Figure S4. The FTIR-ATR spectrum of polymer material PM-0, containing polymer PVC and plasticiser ADO.

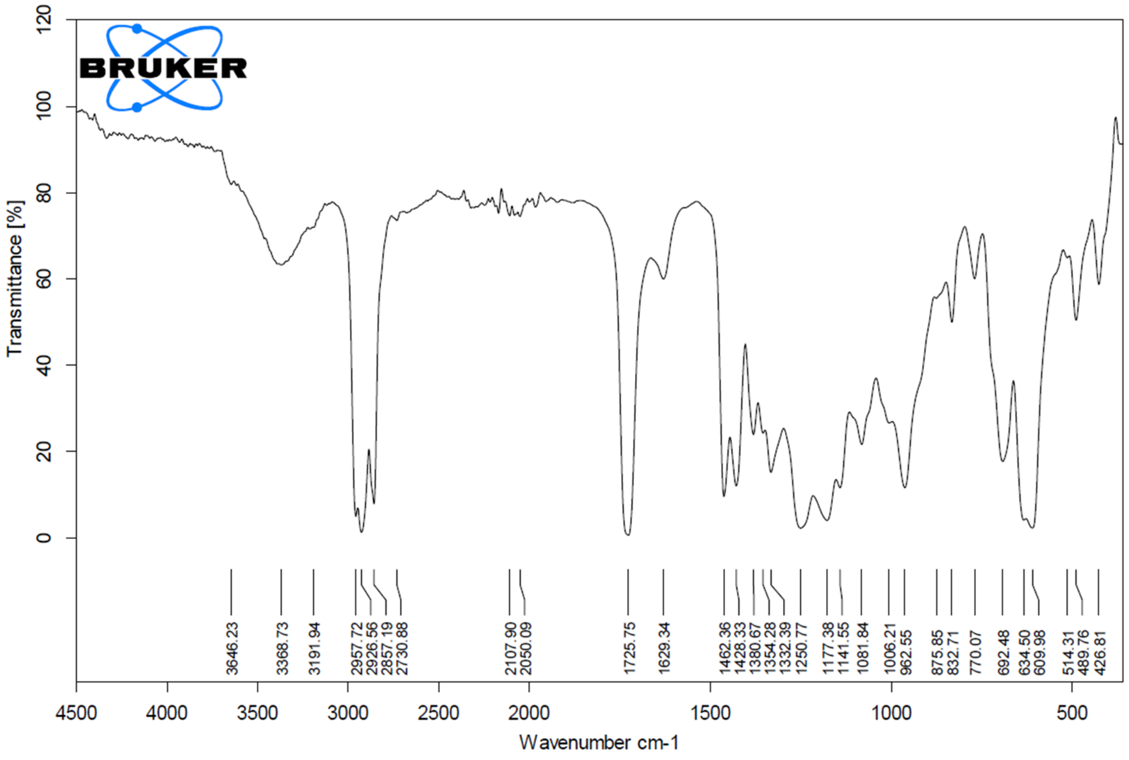

Figure S5. The FTIR-ATR spectrum of polymer material PM-1, containing polymer PVC, plasticiser ADO and DES-1 (composed of Cyphos IL 101 and diacetamide).

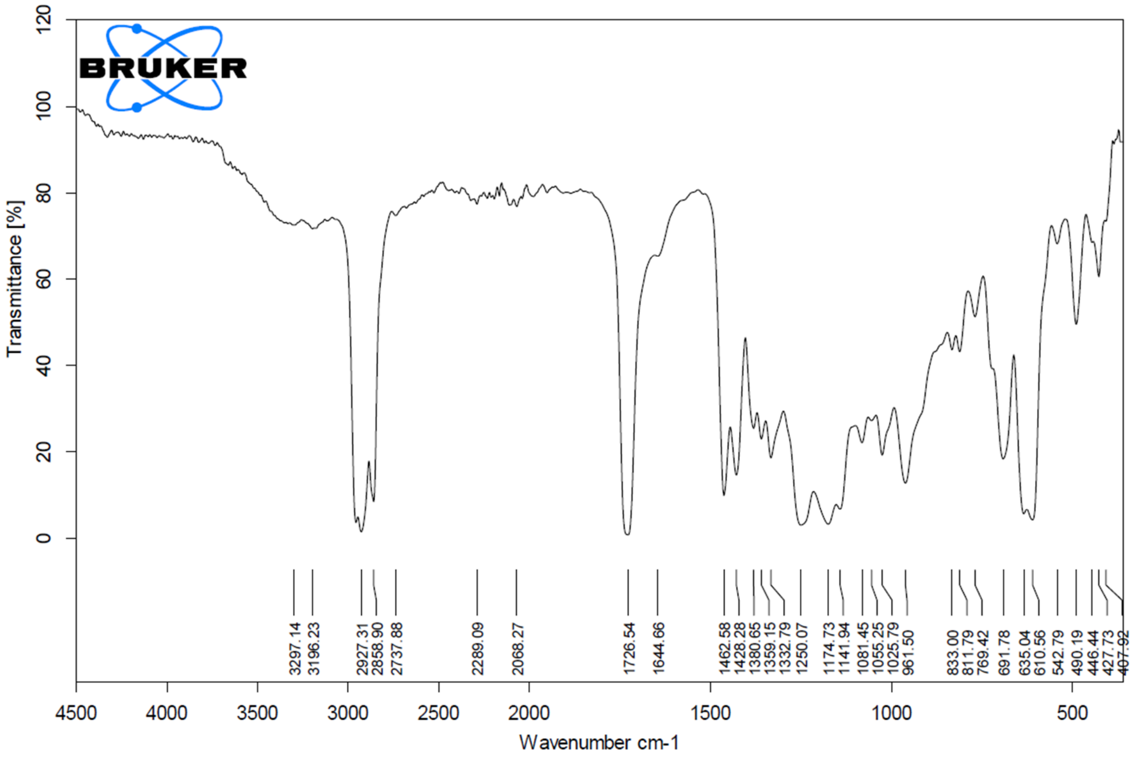

Figure S6. The FTIR-ATR spectrum of polymer material PM-2, containing polymer PVC, plasticiser ADO and DES-2 (composed of Cyphos IL 104 and diacetamide).

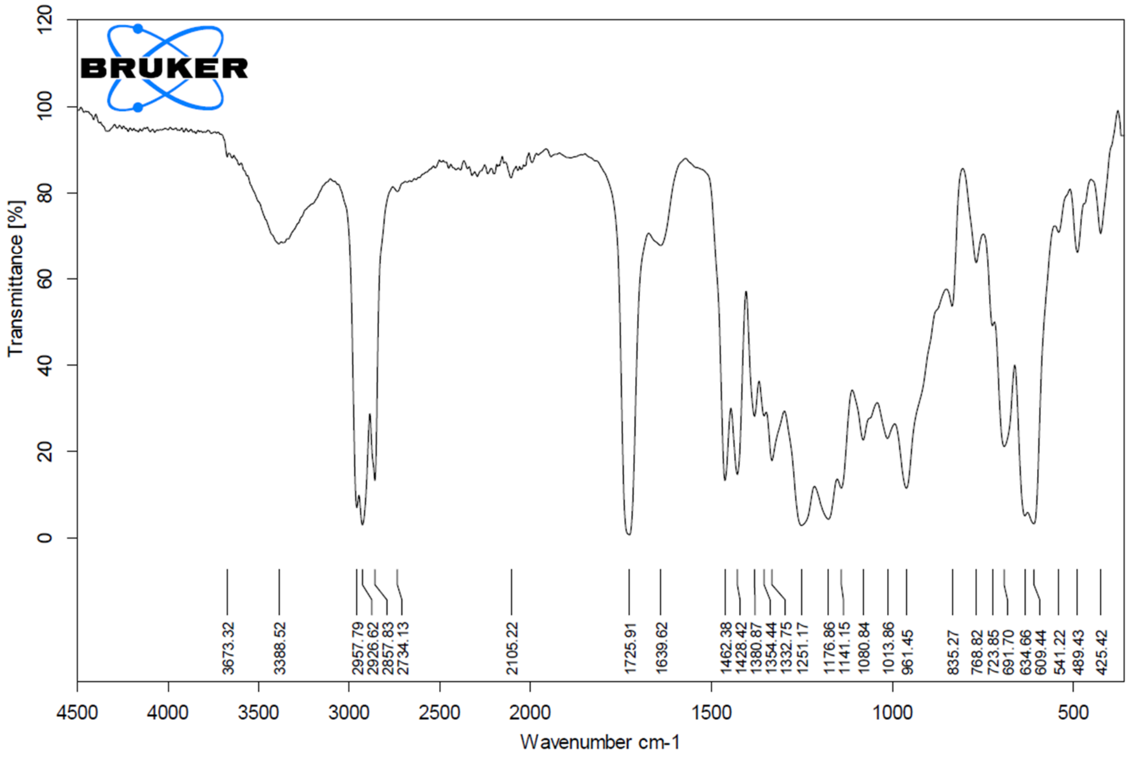

Figure S7. The FTIR-ATR spectrum of polymer material PM-5, containing polymer PVC, plasticiser ADO and DES-3 (composed of Aliquat 336 and diacetamide).

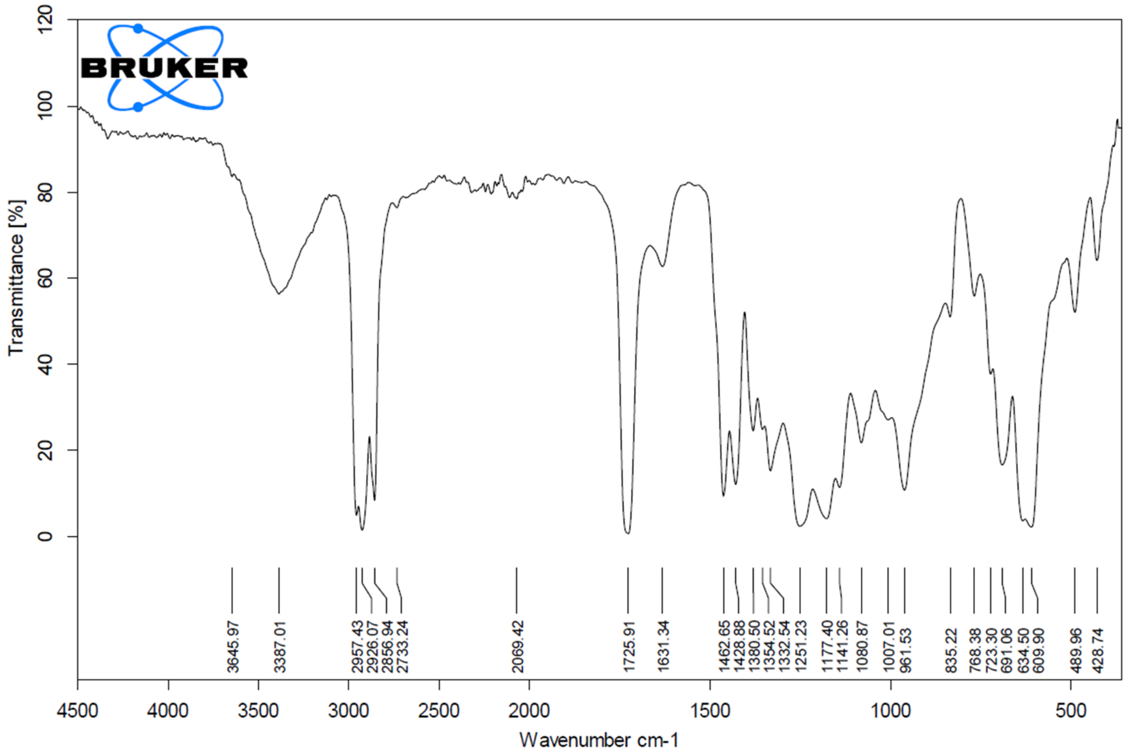

Figure S8. The FTIR-ATR spectrum of polymer material PM-6, containing polymer PVC, plasticiser ADO and ionic liquid Aliquat 336.
